# Cytokine-induced expandable memory NK cells with significant metabolic, epigenetic remodeling, and persistence properties

**DOI:** 10.1038/s41420-026-03260-2

**Published:** 2026-07-21

**Authors:** Hongye Wang, Jingwei Tu, Yi Luo, Xinxin Yuan, Xin Fang, Zhengyi Lu, Li Han, Yongjia Xue, Wencheng Zhang, Qianggang Dong, Zhiying He

**Affiliations:** 1 Institute for Regenerative Medicine, Medical Innovation Center and State Key Laboratory of Cardiology, Shanghai East Hospital, School of Life Sciences and Technology, Tongji University, Shanghai, 200123 China; 2 Shanghai iCELL Biotechnology Co., Ltd, Shanghai, 200335 China; 3 Shanghai Engineering Research Center of Stem Cells Translational Medicine, Shanghai, 200335 China; 4 Shanghai Institute of Stem Cell Research and Clinical Translation, Shanghai, 200120 China; 5 Shanghai Cancer Institute, Shanghai Jiaotong University School of Medicine, Shanghai, 200030 China

**Keywords:** Immunotherapy, Cell therapies

## Abstract

Recent investigations have indicated that NK cells induced overnight with IL-12/15/18 cytokines possess memory-like characteristics (refers as CIML NK, cytokine induced memory-like NK cells), demonstrating enhanced effector function and prolonged persistence. Preclinical studies and very early clinical trials revealed the promising clinical activity of CIML NK cells in the treatment of hematological malignancies, especially acute myelocytic leukemia (AML). However, the current manufacturing method for CIML NK cells do not endow these cells the ability to expand robustly ex vivo. The low production efficiency limits their clinical application. In this study, we established a novel expansion platform for generating memory-like NK cells by first inducing memory properties through stimulation with CD16a, IL-15, and IL-18, followed by a robust expansion phase. This method can effectively and stably expand healthy donor-derived Peripheral NK (PBNK) cells by more than 200-fold within two weeks, with a purity exceeding 95%. Importantly, these ex vivo expanded NK (ExNK) cells exhibited hypomethylated state in the CNS-1 region (Conserved Noncoding Sequence 1) of *IFNG* gene, stronger metabolic ability, and enhanced effector function when compared to CIML NK cells. Based on these features, we named these expanded NK cells but still possess memory-like characteristics as mExNK (memory-like expanded NK cells). Bulk mRNA-seq further uncovered a high similarity between mExNK and CIML NK cells, with both highly expressing genes being related to proliferation, metabolism, and memory. Mouse tumor models proved that the infused mExNK exhibited longer persistence in vivo and stronger effector functions. Taken together, the method described herein showed an enhanced expansion efficiency for human CIML NK cells and would facilitate their clinic applications in cancer therapy.

**Figure:**
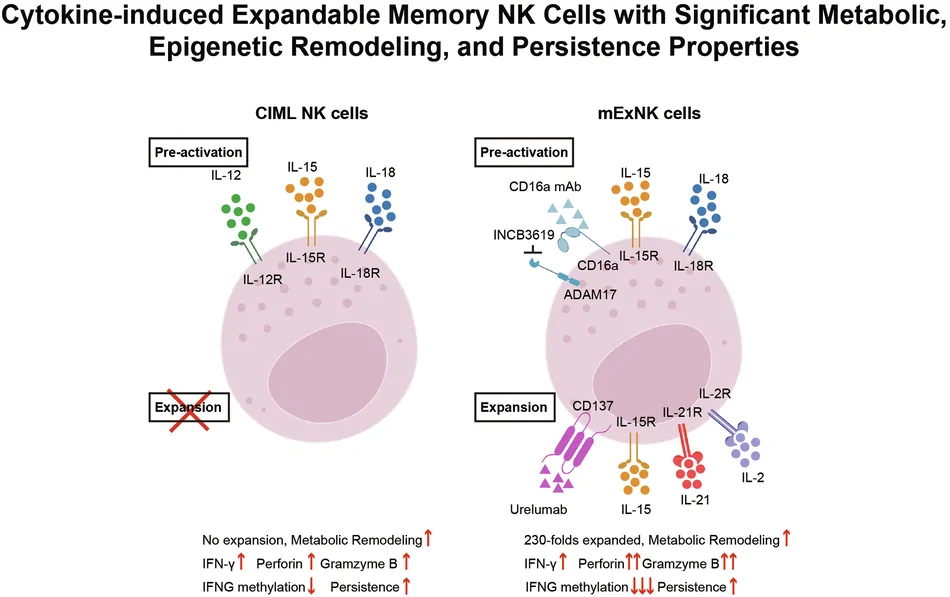

## Background

Natural killer (NK) cells are innate lymphocytes capable of rapidly killing malignant or virally infected cells without prior sensitization [1, 2]. Their activity is regulated by a balance of germline-encoded activating and inhibitory receptors [3]. Upon activation, they eliminate targets directly via perforin and granzyme release or through antibody-dependent cellular cytotoxicity (ADCC) via CD16a. This profile makes NK cells attractive for adoptive cell therapy (ACT), noted for a favorable safety profile even from allogeneic donors [4, 5]. While this NK cell-mediated immunity holds great promise in cancer therapy, challenges remain, including a limited persistence and functionality in vivo, which would compromise their clinical effectiveness [6].

In the last two decades, accumulating evidence have revealed that the NK cells possess heterogeneous memory properties that can be broadly categorized into three distinct pathways [7]: (i) cytokine-induced memory-like (CIML) differentiation, which is antigen-independent and driven solely by cytokine pre-activation; (ii) antigen-specific adaptive NK cell memory (exemplified by hapten-specific hepatic NK) [8–10], and (iii) antigen-nonspecific trained immunity in NK cells [11]. In humans, the CIML NK cells, pre-activated with IL-12, IL-15, and IL-18, trigger metabolic reprogramming (a shift to aerobic glycolysis) and stable epigenetic changes (e.g., DNA methylation at the *IFNG* gene) [12–16]. The synergistic coordination between the metabolic and epigenetic reprogramming described above, and among the others, composed the molecular basis of NK cell memory [15]. Upon re-encountering tumor targets or cytokines, CIML NK cells demonstrate markedly enhanced IFN-γ production and effector functions [17, 18]. They can persist for several months in both clinical patients and mouse models, significantly longer than conventional NK cells [19–21]. Multiple clinical trials (NCT02782546, NCT03068819, NCT04024761, NCT04290546, NCT04354025, and NCT04634435, from *clinicaltrials.gov*) have been initiated with CIML NK cells, demonstrating notable safety and efficacy in treating AML patients.

However, the current preparation methods for CIML NK cells used in these trials lack effective cell expansion, limiting their broad clinical application. The growing clinical demand for large quantities of CIML NK cells urgently requires the development of more efficient and convenient methods for their ex vivo expansion.

To address this, we developed a novel, feeder-free expansion platform. It employs an initial activation phase using CD16a antibody plus IL-15/IL-18 to induce the development of immune memory, followed by a high-efficiency expansion phase using IL-2, IL-15, and IL-21 to maintain memory properties. This method achieved over 200-fold NK cell expansion in 14 days. We then systematically and rigorously validated the immune memory attributes of these expanded NK cells compared with CIML NK and PBNK cells across five dimensions: phenotype, metabolism, epigenetics, function, and persistence. Thus, we named these expanded but still possess memory-like characteristics NK cells as mExNK. The scalable expansion of mExNK cells would pave a new avenue for their clinical applications.

## Results

### Effective expansion of human NK cells with the feeder-free system

**Fig. 1:**
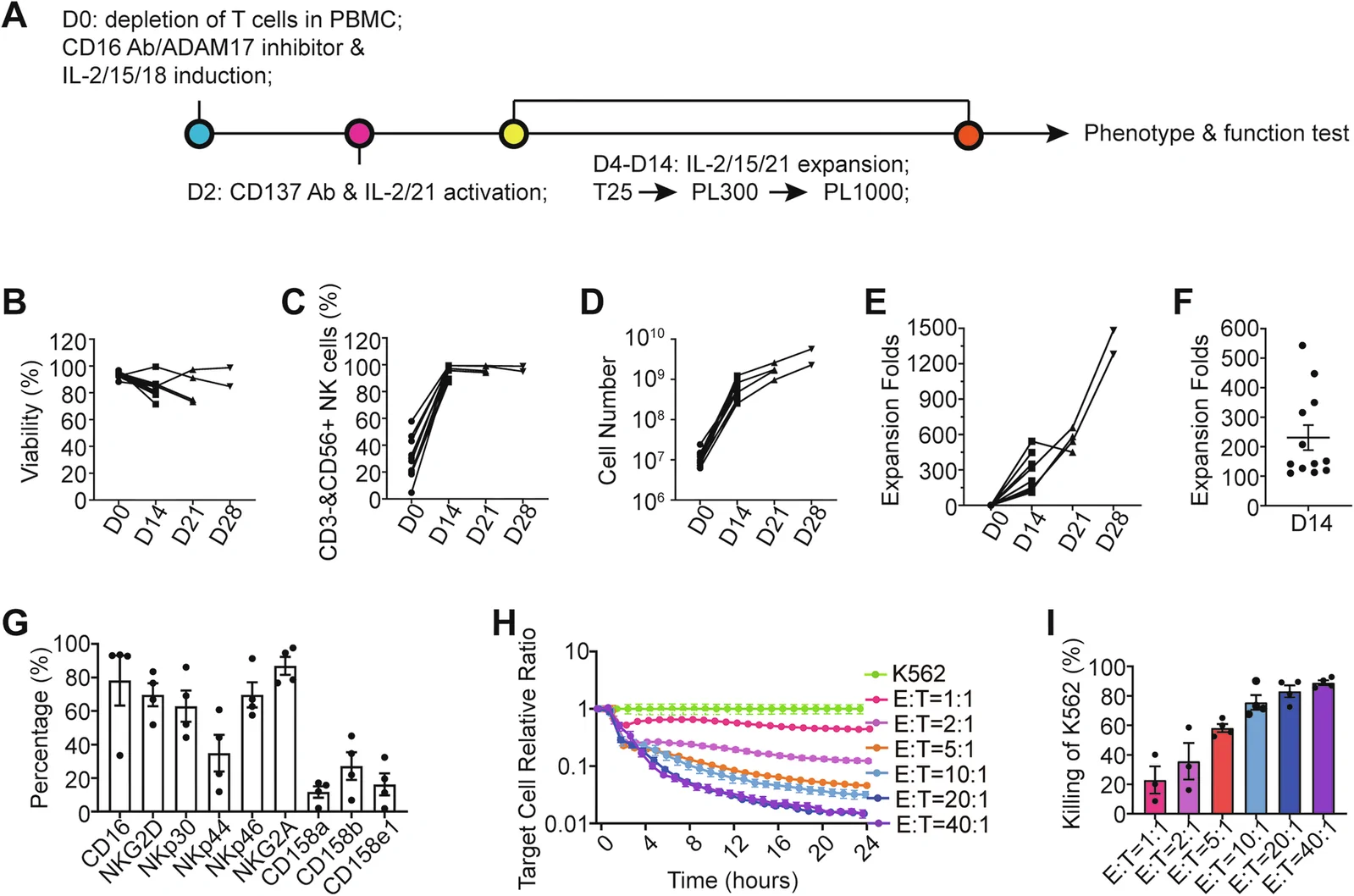
Establishment of a high-purity feeder-free expansion system for human peripheral natural killer cells. **A** Schematic diagram of human PBNK cell activation and expansion. **B** Viability of human PBNK cells during expansion for 28 days. Cells were collected weekly for 4 weeks and analyzed by AOPI staining (*n* = 12). **C** Purity of NK cells identified by flow cytometry of CD3-&CD56+ during expansion period (*n* = 12). **D** Total cell number at different time points over expansion period (*n* = 12). **E** Expansion fold change of human NK cells from all 12 donors (*n* = 12). A total 1380-fold increase of human PBNK cell numbers were obtained by day 28 (4×10^9^) in this expansion system. **F** Average expansion fold change of human NK cells in D14 (*n* = 12). **G** The percentage of human NK cells that expressed NK activating receptors (CD16, NKG2D, NKp30, NKp44, NKp46) and NK inhibitory receptors (NKG2A, CD158a, CD158b, CD158c), respectively, from the flow cytometry assay (*n* = 4). **H** Time-dependent green intensity curves for cultures of the human expanded NK cells and K562 cells (target cells) at the indicated effector cell: tumor cell ratio (E:T) in 24-h Incucyte-based function assay (*n* = 3). K562 cells only was served as the control. Each time point represented the mean of three replicates and the standard error of the mean (SEM) is represented by the error bars. **I** Statistical analysis of relative specific tumor cell lysis in 4-h co-culture from Incucyte-based function assay (*n* = 3-4). Each time point represented the mean of three or four independent experiments from different donors and the SEM is represented by the error bars.

While unstimulated NK cells exhibit limited antitumor efficacy in adoptive cell therapy, multiple activation and expansion protocols have been developed to enhance their cytotoxic potential [22–24]. To prevent T cell overgrowth in final products, we first depleted CD3^+^ T cells from PBMCs. Building on evidence from CIML NK studies, combinations of CD16a antibody with cytokines such as IL-12, IL-15, and IL-18 can promote memory-like properties [16, 25], we stimulated NK cells with CD16a antibody plus IL-15/IL-18 during the first two days (Fig. 1A). Since NK cell activation upregulates the metalloprotease ADAM17, which cleaves surface receptors including CD16a [26, 27], the ADAM17 inhibitor INCB3619 was added concurrently to preserve CD16a expression. CD137 antibody and IL-21, recognized as strong proliferative signals for NK cells [28–30], were subsequently introduced to drive expansion (Fig. 1A). From day 4 onward, IL-2, IL-15, and IL-21 were supplemented to sustain long-term culture. Using this feeder-free system, we consistently obtained expanded NK (ExNK) cells with high viability (>80%) and purity (≥95%) (Fig. 1B–D). Across 12 healthy donors, the mean expansion at two weeks was ~230-fold (range from 110 to 543), with some donors sustaining growth for up to four weeks (Fig. 1E, F).

Flow cytometry revealed that ExNK cells expressed high levels of activating receptors (CD16, NKG2D, NKp30, NKp44, NKp46) and inhibitory receptors NKG2A and CD158b (Fig. 1G). Real-time cytotoxicity assays against K562 targets showed that at 4 h, ExNK cells mediated about 20% killing at a 1:1 E:T ratio, increasing to 80% at 10:1 (Fig. 1H, I).

In summary, we established a feeder-free expansion system that generates highly pure (≥95%), functionally active NK cells expressing multiple activating receptors and exhibiting potent tumor-killing capacity. Although the early activation phase included CD16 antibody and IL-15/IL-18 to induce memory-like features, whether such properties persist after two weeks of expansion requires further investigation.

### ExNK cells showed apparent memory-like properties as determined by phenotype, metabolism and epigenetic remodeling

**Fig. 2:**
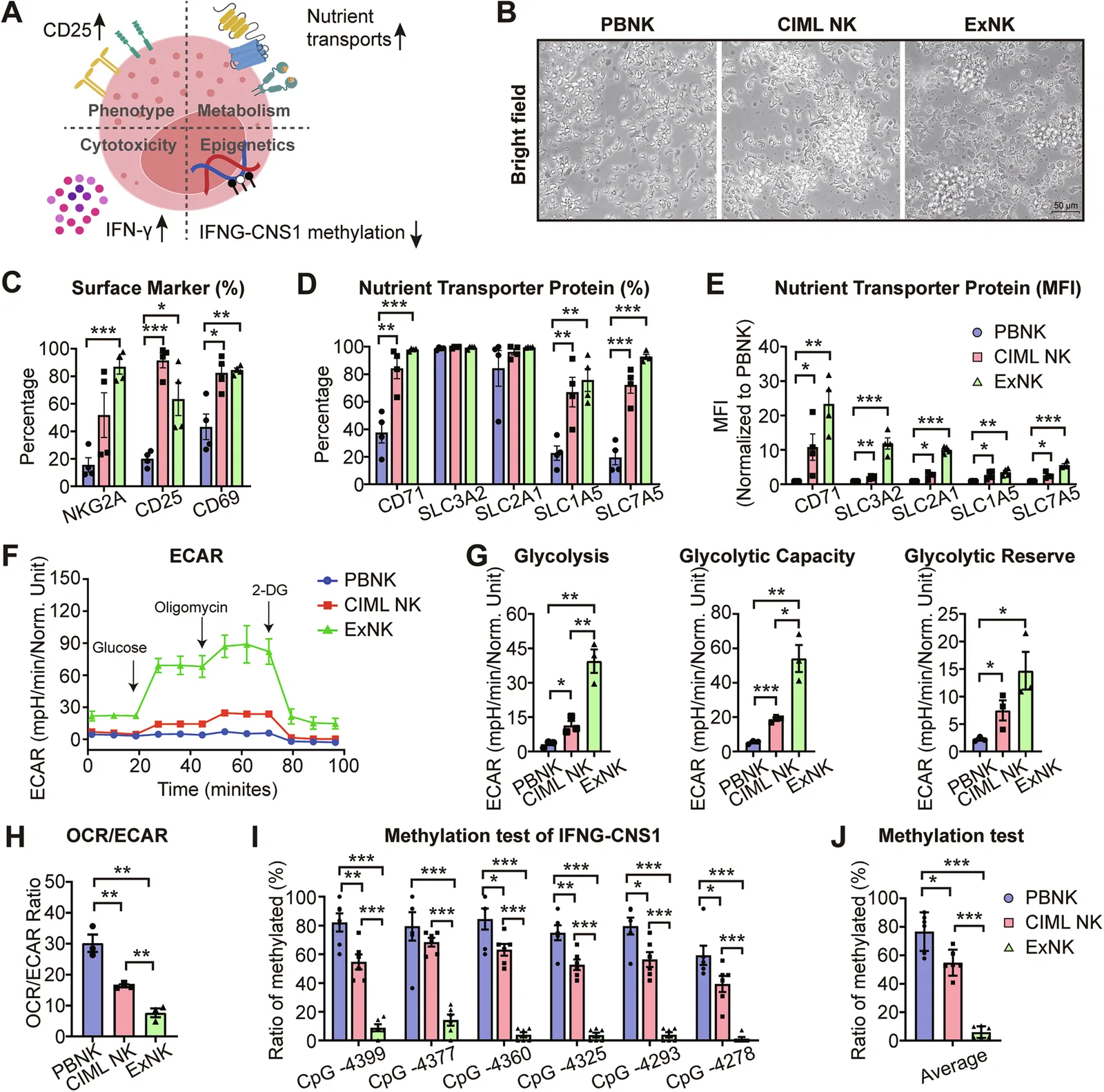
ExNK cells possess typical memory-like properties characterized by phenotypic, metabolic and epigenetic features. **A** Schematic representation of the features observed in CIML NK cells. **B** Bright field images of the PBNK, CIML NK and ExNK cells. Scale bars, 50 µm. **C** Positive percentage of cells expressing surface markers (CD25, CD69, and NKG2A) in PBNK, CIML NK and ExNK by flow cytometry assay (*n* = 4). *p < 0.05, **p < 0.01, ***p < 0.001. **D, E** Positive percentage and mean fluorescent intensity (MFI) for the cells expressing nutrient transport proteins (CD71, SLC3A2, SLC2A1, SLC1A5 and SLC7A5) in PBNK, CIML NK and ExNK samples measured by flow cytometry (*n* = 4). *p < 0.05, **p < 0.01, ***p < 0.001. **F** Seahorse assay showing the extracellular acidification rate (ECAR) profile in PBNK, CIML NK and ExNK cells, respectively (*n* = 3). All samples were normalized to cell number. **G** Quantification of ECAR in glycolysis, glycolytic capacity, and glycolytic reserve (*n* = 3). Graphs represented three independent experiments from different donor and the SEM is represented by the error bars. *p < 0.05, **p < 0.01, ***p < 0.001. **H** The OCR/ECAR ratios was assessed by analyzing the basal energy metabolism of PBNK, CIML NK and ExNK cells (*n* = 3). **p < 0.01. **I** DNA methylation status of six CpG sites within the CNS-1 region of the *IFNG* gene (located at −4399, −4377, −4360, −4325, −4293, and −4278 relative to the transcription start site) was analyzed by conventional bisulfite-sequencing from PBNK, CIML NK and ExNK cell samples (*n* = 6). *p < 0.05, **p < 0.01, ***p < 0.001. **J** Average DNA methylation level of six CpG sites in panel G (*n* = 6). *p < 0.05, ***p < 0.001.

Prior studies have shown that CIML NK cells possess a distinct phenotype, metabolic profile, and epigenetic state underlying their enhanced function (Fig. 2A) [15, 16, 31]. Here, we systematically evaluated our ExNK cells against these established CIML benchmarks.

PBNKs cultured in maintenance medium served as a negative control. CIML NK cells were generated by brief IL-12/15/18 activation followed by rest in maintenance medium served as a positive control. Morphologically, both ExNK and CIML NK cells formed prominent clusters, unlike PBNKs (Fig. 2B).

CIML NK cells upregulate CD25 (IL-2Rα), enabling formation of high-affinity IL-2 receptors and supporting robust proliferation and cytotoxicity even under low IL-2 conditions [32]. Flow cytometry revealed that ExNK and CIML NK cells similarly upregulated CD25 and CD69 compared to PBNKs, while expression of other receptors was comparable (Fig. 2C and Supplementary Fig. 1A).

Metabolically, CIML NK cells exhibit a pronounced glycolytic shift, accompanied by increased expression of nutrient transporters such as GLUT1 (SLC2A1), amino acid transporters (SLC1A5, SLC7A5, SLC3A2/CD98), and CD71 (transferrin receptor) [33, 34]. Both ExNK and CIML cells showed elevated levels of these transporters (Fig. 2D, E). Functional metabolic assays confirmed that both ExNK and CIML NK cells exhibited enhanced glycolysis, glycolytic capacity, and glycolytic reserve relative to PBNKs (Fig. 2F, G), though oxidative phosphorylation (OXPHOS) parameters (including basal respiration, spare respiratory capacity, and ATP production) remained unchanged across all groups (Supplementary Fig. 1B, C). The OCR:ECAR ratio, reflecting a shift toward glycolytic metabolism [35], was similarly reduced in both ExNK and CIML NK cells (Fig. 2H).

A hallmark of CIML NK cells is epigenetic reprogramming at the *IFNG* locus [13, 36–38]. Analysis of six CpG sites in the conserved noncoding sequence‑1 (CNS‑1) of *IFNG* gene showed profound hypomethylation in ExNK cells (average 6% methylation), comparable to CIML NK cells (54.8%) and distinctly lower than PBNKs (76.7%) (Fig. 2I, J and Supplementary Fig. 1D, E). To further investigate this epigenetic reprogramming, we performed a time‑course analysis using donor‑matched mExNK and CIML NK cells. Methylation levels progressively declined over time, with mExNK cells achieving significantly lower methylation than CIML NK cells by day 5, and this reduction persisted through day 14 (Supplementary Fig. 2A–E). Thus, although epigenetic reprogramming is time‑dependent, the unique factors of the mExNK expansion system actively promote and sustain lower methylation levels.

In summary, ExNK cells, akin to CIML NK cells but to an even greater extent, undergo phenotypic, metabolic, and epigenetic reprogramming that clearly distinguishes them from PBNKs. We therefore designate these cells as memory-like expanded NK (mExNK) cells.

### mExNK had potent effector function and persistence ability in vitro

**Fig. 3:**
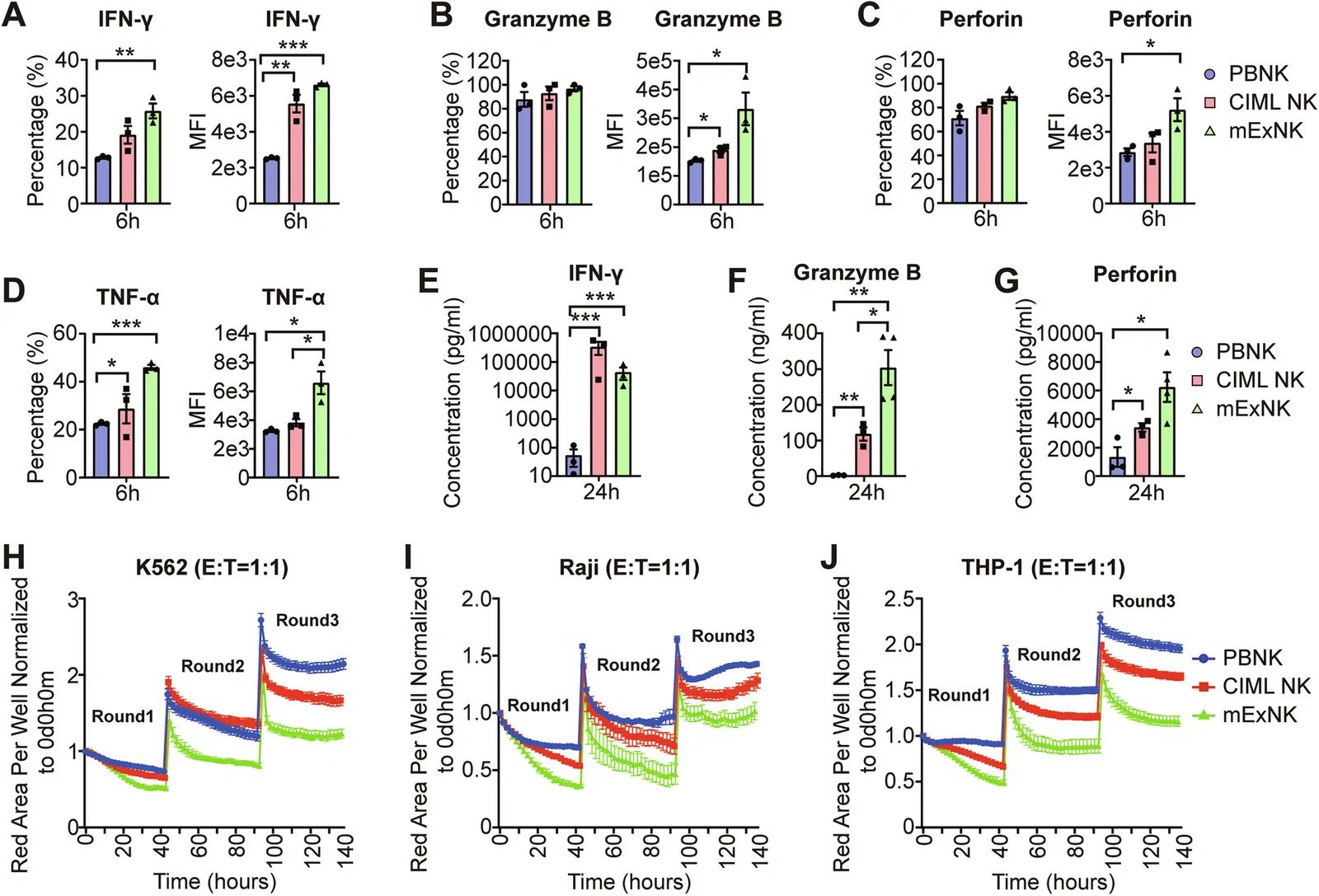
In vitro cytotoxicity activities and persistence ability of mExNK cells. **A–D** The positive percentage and MFI of cells expressing IFN-γ **A**, Granzyme B **B**, Perforin **C**, and TNF-α **D** in PBNK, CIML NK and mExNK samples measured by flow cytometry (*n* = 3). Left panels for each set present positive percentage, right panels are for MFI. * *p* < 0.05, ** *p* < 0.01, *** *p* < 0.001. **E–G** The secretion/production of IFN-γ **E**, Granzyme B **F**, Perforin **G** was assessed by ELISA (enzyme-linked immunosorbent assay) of the culture supernatants by co-culture of PBNK, CIML NK or mExNK cells with target K562 cells for 24 h (*n* = 3-4). **H–J** Data were generated with Incucyte. Cumulative data from three repeated-round tumor rechallenge assay showing K562 **H**, Raji **I**, and THP-1 **J** viability, quantified by relative red intensity, in each co-culture condition with 1:1 (E:T) ratio over the course of 140 h (*n* = 3). Data are shown as mean ± SEM of 3 replicates per timepoint.

Having established that mExNK cells acquire memory-like attributes, we next assessed whether this translates to enhanced effector functions. To test this, we first evaluated cytokine expression following a 6-h co-culture with K562 target cells (E:T ratio=1:1). Flow cytometric analysis revealed that both mExNK and CIML NK cells produced significantly higher levels of IFN-γ, TNF-α, and Granzyme B than PBNKs, with mExNK cells showing the most robust increase (Fig. 3A, B, D). Perforin expression was also elevated in mExNK compared to PBNKs, though not significantly different from CIML NK (Fig. 3C). These results were confirmed by ELISA after 24-h, with mExNK and CIML NK cells secreting substantial more IFN-γ, Granzyme B, and perforin than PBNKs (Fig. 3E–G).

Tumor rechallenge assay can be coupled with profiling immune cell activation, exhaustion and persistence phenotypes, provide a more accurate reflection of the in vivo anti-tumor capacity of immune cells [39]. Here we developed a rechallenge model to assess serial killing capacity under high tumor burden. NK cells were co-cultured with tumor lines (K562, Raji, THP-1) at E:T ratios of 1:1 or 1:2, and fresh labeled tumor targets were replenished every two days over three rounds (6 days). mExNK cells consistently exhibited stronger and more sustained tumor killing than both CIML NK and PBNK cells, with differences emerging as early as 6–8 h and maintained throughout the assay (Fig. 3H–J and Supplementary Fig. 3A–I).

Given that mExNK cells express high levels of NKG2A, a receptor known to inhibit the killing of HLA-E+ tumor cells and promote tumor immune evasion [40, 41], we generated HLA-E-overexpressing K562 cells, which increased surface HLA-E expression from approximately 5% to 40% (Supplementary Fig. 4A, B). Cytotoxicity assays revealed that mExNK cells exhibited significantly reduced killing of HLA-E-overexpressing K562 cells. CIML NK cells also showed decreased killing, whereas PBNK cells, which express low levels of NKG2A, remained unaffected (Supplementary Fig. 4C).

In summary, mExNK cells not only exhibit enhanced cytokine production but also possess superior persistence and serial killing capacity compared to CIML NK cells.

### The mExNK cells display an advanced effector and activation gene expression, but similar memory profiles with CIML NK

**Fig. 4:**
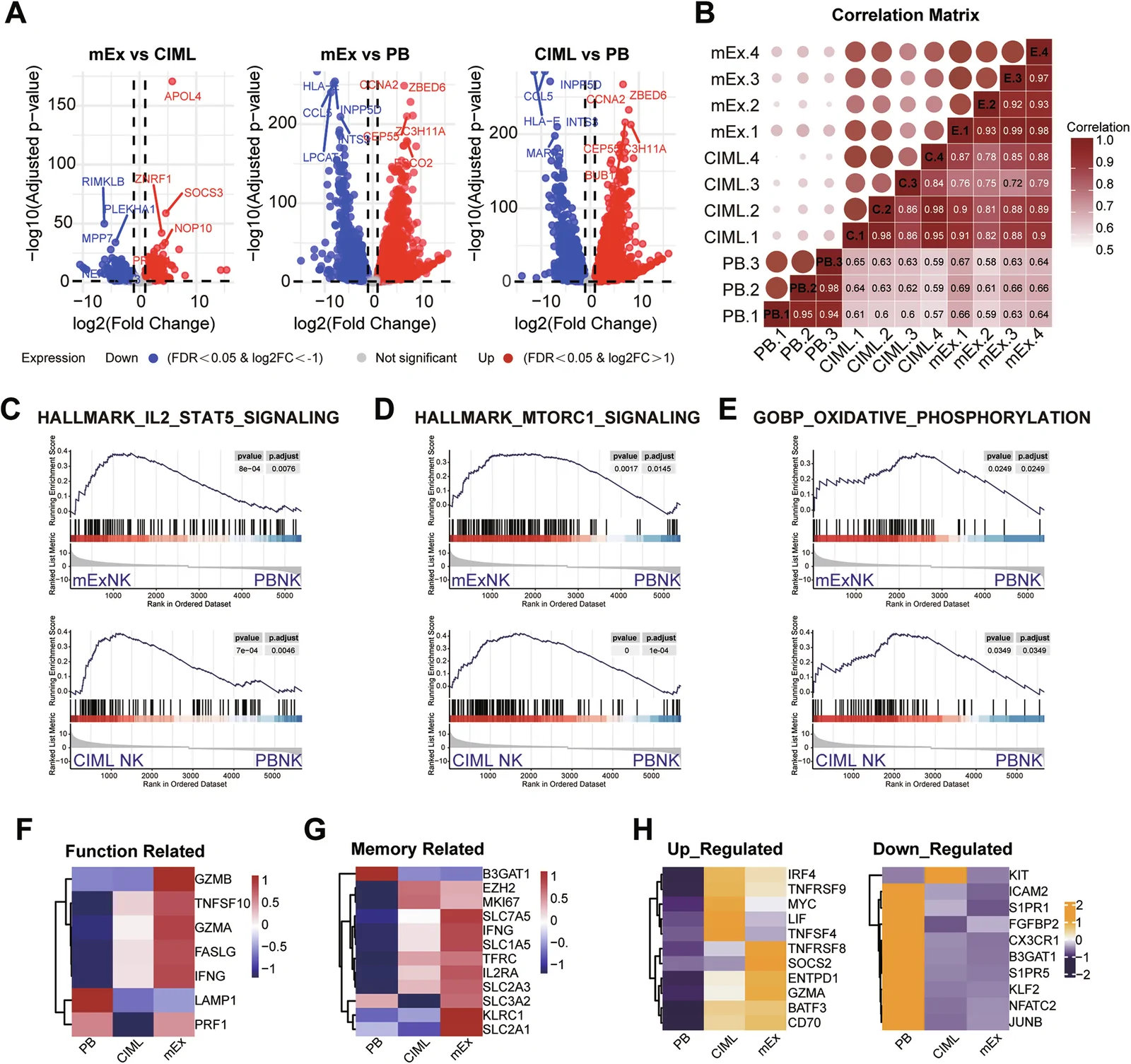
mRNA-sequencing analysis of PBNK, CIML NK and mExNK samples. **A** Distribution of the differentially expressed genes (DEGs) shown as volcano plots between mEx and CIML, mEx and PB, CIML and PB group (*n* = 3-4). DEGs were screened according to the criteria of FDR<0.05, and |log2FC|>1. Down-regulated genes are marked with blue and up-regulated genes with red. **B** Correlation analysis between the samples. PBNK group, *n* = 3; CIML NK group, *n* = 4; mExNK group, *n* = 4. **C–E** Gene set enrichment analysis (GSEA) enrichment plot for the IL2 STAT5 signaling **C**, mTORC1 signaling **D**, and oxidative phosphorylation **E** pathways between mExNK and PBNK or CIML NK and PBNK group. Top panel indicated mExNK vs PBNK group, bottom panel indicated CIML NK vs PBNK group. The y-axis represented enrichment score (ES) and the x-axis showed genes (vertical black lines) represented in different pathways. The colored band at the bottom represented the degree of correlation of the expression of these genes (red for a high gene expression and blue for a low gene expression). **F, G**. Hierarchical clustering heatmap of partial genes associated with NK function related and memory-related functions. Horizontal axis indicated cluster analysis of PBNK, CIML NK and mExNK groups. Vertical axis showed analysis of selected genes. Red represented upregulated DEGs, blue represented downregulated DEGs, and white represented the genes expression with no difference. **H** Hierarchical clustering heatmap of up-regulated and down-regulated genes reported in the related paper.

Bulk mRNA sequencing revealed that mExNK and CIML NK cells share a highly similar transcriptional landscape, distinct from PBNKs. Far fewer genes were differentially expressed (DEGs) between mExNK and CIML NK cells than between either population and PBNKs, and their gene expression profiles correlated strongly (Fig. 4A, B and Supplementary Fig. 5A). Over 70% of DEGs (both up- and down-regulated) compared to PBNKs were shared between mExNK and CIML NK cells, and Gene Ontology (GO) analysis showed common enrichment in pathways such as DNA replication and cellular metabolism (Supplementary Fig. 5B, 5C, 5F).

Despite this core similarity, mExNK cells possessed a unique transcriptional signature. Direct comparison with CIML NK cells identified enrichment in pathways governing T cell activation, cell-cell adhesion, and cytokine regulation (Supplementary Fig. 5D), which aligns functionally with their superior performance in serial tumor rechallenge assays (Supplementary Fig. 5E).

Gene Set Enrichment Analysis (GSEA) further confirmed shared upregulation of hallmark pathways (including E2F targets, G2M checkpoint, IL2-STAT5 signaling, and mTORC1 signaling) in both mExNK and CIML NK cells versus PBNKs (Supplementary Fig. 5G–I). Given the established roles of IL2‑STAT5 and mTORC1 pathways in NK cell survival, metabolism, and function [42, 43], we focused subsequent validation on these, along with oxidative phosphorylation (OXPHOS). GSEA confirmed that mExNK and CIML NK cells exhibited significant upregulation of all three pathways relative to PBNK (Fig. 4C–E).

Notably, mExNK cells maintained higher expression of key effector genes, including *TNFSF10* (TRAIL), *FASLG* (FASL), *GZMA*, *GZMB*, and *IFNG* (Fig. 4F). Both mExNK and CIML NK cells highly expressed *KLRC1* (NKG2A), *IL2RA* (CD25), and genes encoding nutrient transporters such as *TFRC* (CD71), *SLC1A5*, and *SLC7A5* (Fig. 4G), consistent with our phenotypic and metabolic data (Fig. 2C–E).

Transcriptomic profiling also revealed elevated expression of *EZH2* and *MKI67*, alongside low expression of *B3GAT1* (CD57), in both mExNK and CIML NK cells (Fig. 4G). This profile matches with a previously reported CD57-NKG2A+ EZH2+ MKI67+ CIML NK subset associated with rapid cycling and enhanced trained immunity [44]. Furthermore, we observed upregulation of activation-related genes (e.g., *IRF4*, *TNFRSF9*, *MYC*) and downregulation of maturation- and adhesion-related genes (e.g., *FGFBP2*, *KLF2*, *CX3CR1*), mirroring recently reported memory-like NK transcriptional signatures [45] (Fig. 4H).

In summary, mExNK cells share a core memory-like transcriptional program with CIML NK cells but also display a distinct gene expression profile linked to adhesion and effector function which is consistent with their enhanced and sustained anti-tumor activity.

### mExNK cells showed efficacy against THP‑1 leukemia tumors and persist long‑term in vivo

**Fig. 5:**
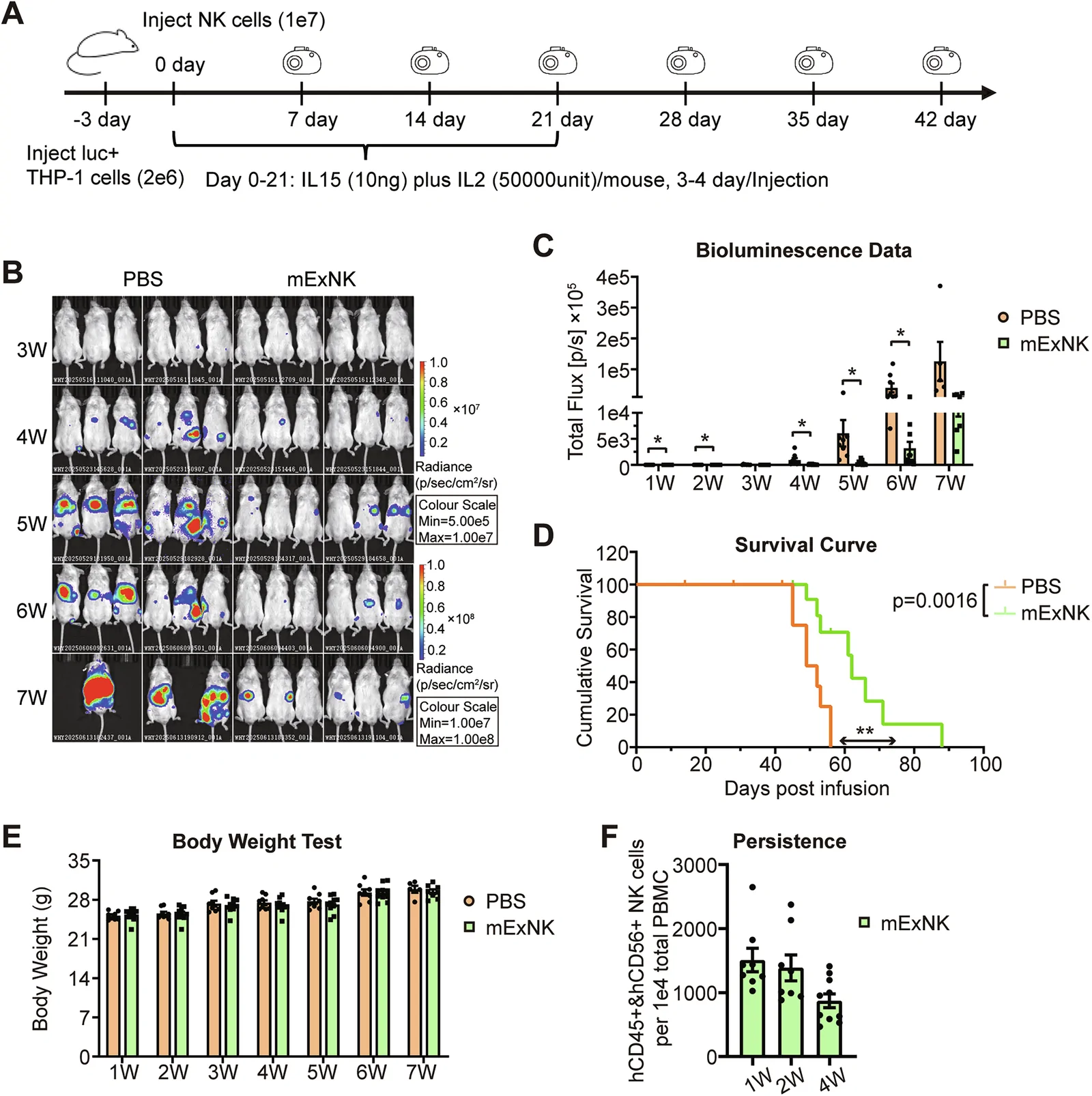
In vivo antitumor activities of mExNK cells against THP-1 cells in NSG mouse model. **A** Schematic diagram of experimental design, including THP-1-luc+ tumor cell transplantation, mExNK cell treatment, cytokine administration, and bioluminescence imaging of xenografted tumors. **B, C** mExNK cells were effective in reducing tumor progression of THP-1 cells. Shown are representative bioluminescence images (B) and quantification **C** of THP-1-derived leukemia tumor in NSG mice treated with mExNK cells, or vehicle control (*n* = 8–10). Quatification results are shown as mean ± SEM. *p < 0.05, **p < 0.01, ***p < 0.001. **D** mExNK cells were effective in prolonging the survival of leukemia tumor-bearing mice. Shown are survival curves of NSG mice transplanted with THP-1 and treated with mExNK cells, or vehicle control (*n* = 8–10). **p < 0.01. **E** mExNK cells were safe with no apparent toxic side effects. The body weight tests were shown for NSG mice that have been transplanted with THP-1 and treated with mExNK cells or control animals (*n* = 8-10). **F** Long-term persistence of mExNK cells in the leukemia tumor-bearing mice identified by flow cytometry of hCD45+ &hCD56+ NK cells in mice peripheral blood (*n* = 6–10).

We next assessed the in vivo anti-tumor efficacy and persistence of mExNK cells using an NSG mouse model engrafted with THP-1-Luc+ human leukemia cells [46]. Mice received tumor cells intravenously, followed three days later by an infusion of mExNK cells, control mice received tumor cells only. All mice received supportive cytokine injections (IL-2/IL-15) post-transfer (Fig. 5A). mExNK cell treatment significantly inhibited tumor growth and extended survival compared to controls, without causing apparent toxicity as indicated by stable body weight (Fig. 5B–E). To evaluate persistence, we measured human NK cells (hCD45+hCD56+) in peripheral blood over time. Although their numbers declined weekly, mExNK cells remained detectable even four weeks after infusion (Fig. 5F).

To further benchmark the efficacy of mExNK cells, we compared them to NK cells expanded using a commercial kit (hereafter termed cExNK, conventional expanded NK). While cExNK cells met baseline quality (viability/purity > 80%), their average 2-week expansion (~40-fold) was markedly lower than that of mExNK cells (Supplementary Fig. 6A–D). Phenotypically, cExNK cells lacked high CD25 expression, a hallmark of memory-like cells (Supplementary Fig. 6E). Functionally, they showed reduced expression of key nutrient transporters and secreted lower levels of IFN-γ, Granzyme B, and Perforin compared to mExNK cells (Supplementary Fig. 6F–I). Transcriptomic analysis revealed profound differences that over 4200 genes were differentially expressed between cExNK and mExNK cells, far more than between mExNK and CIML NK cells (1149 genes) (Supplementary Fig. 6J). cExNK cells were notably deficient in pathways related to immune response and metabolism (Supplementary Fig. 6K), with downregulation of critical effector genes (*IFNG*, *GZMB*), transporters (*SLC7A5*, *SLC1A5*), and transcription factors (*STAT1*, *STAT3*, *RUNX1*) (Supplementary Fig. 6L). Collectively, these multi-faceted results demonstrate that mExNK cells possess superior properties not only relative to CIML NK cells but also, and more profoundly, to conventionally expanded NK cells.

### mExNK cells exhibit a favorable safety profile

Furthermore, we assessed the safety profile of mExNK cells using in vitro cytokine release syndrome (CRS) and graft-versus-host disease (GvHD) assays [47, 48]. Consistent with the established favorable safety profile of NK cell-based therapies [49], no detectable differences in IL-6, IFN-γ, or TNF-α levels were observed among mExNK, CIML NK, and PBNK cells when co-cultured with K562 tumor cells and monocytes, except that PBNK cells secreted significantly higher IL-6 (Supplementary Fig. 7A–C). Furthermore, similar levels of IL-6, IFN-γ, and TNF-α were observed between T cells and mExNK cells, even though monocytes and T cells were derived from the same donor to avoid alloreactivity, whereas mExNK cells were not derived from the same donor (Supplementary Fig. 7A–C).

To evaluate the potential GvHD activity, allogeneic PHA-blasts, autologous PHA-blasts, and K562 tumor target cells were separately co-cultured with mExNK cells for cytotoxicity assessment. T cells, CIML NK and PBNK cells were used as controls. Interestingly, mExNK cells exhibited potent killing activity against K562 cells but showed minimal cytotoxicity against both allogeneic and autologous PHA-blasts (Supplementary Fig. 7D–F). In contrast, PBNK and CIML NK cells displayed higher killing activity against allogeneic and autologous PHA-blasts, with PBNK cells showing the strongest effect. Taken together with the cytokine secretion profiles, these results suggest that although mExNK cells produce relatively high levels of granzyme B and IFN-γ, they do not attack autologous or allogeneic normal cells; instead, they preferentially target and kill tumor cells (Supplementary Fig. 7G–I).

In summary, our findings further validate that mExNK cells possess superior tumor-killing capacity while maintaining a high safety profile.

### IL-21 acts in synergy with IL-2 to promote memory NK cell expansion ex vivo

To define the mechanisms enabling memory-like NK cell expansion, we analyzed their transcriptional profiles. Although both CIML NK and mExNK cells upregulated genes linked to cell proliferation (e.g., DNA replication), IL-12/15/18 stimulation alone fails to drive substantial expansion, limiting clinical scalability.

**Fig. 6:**
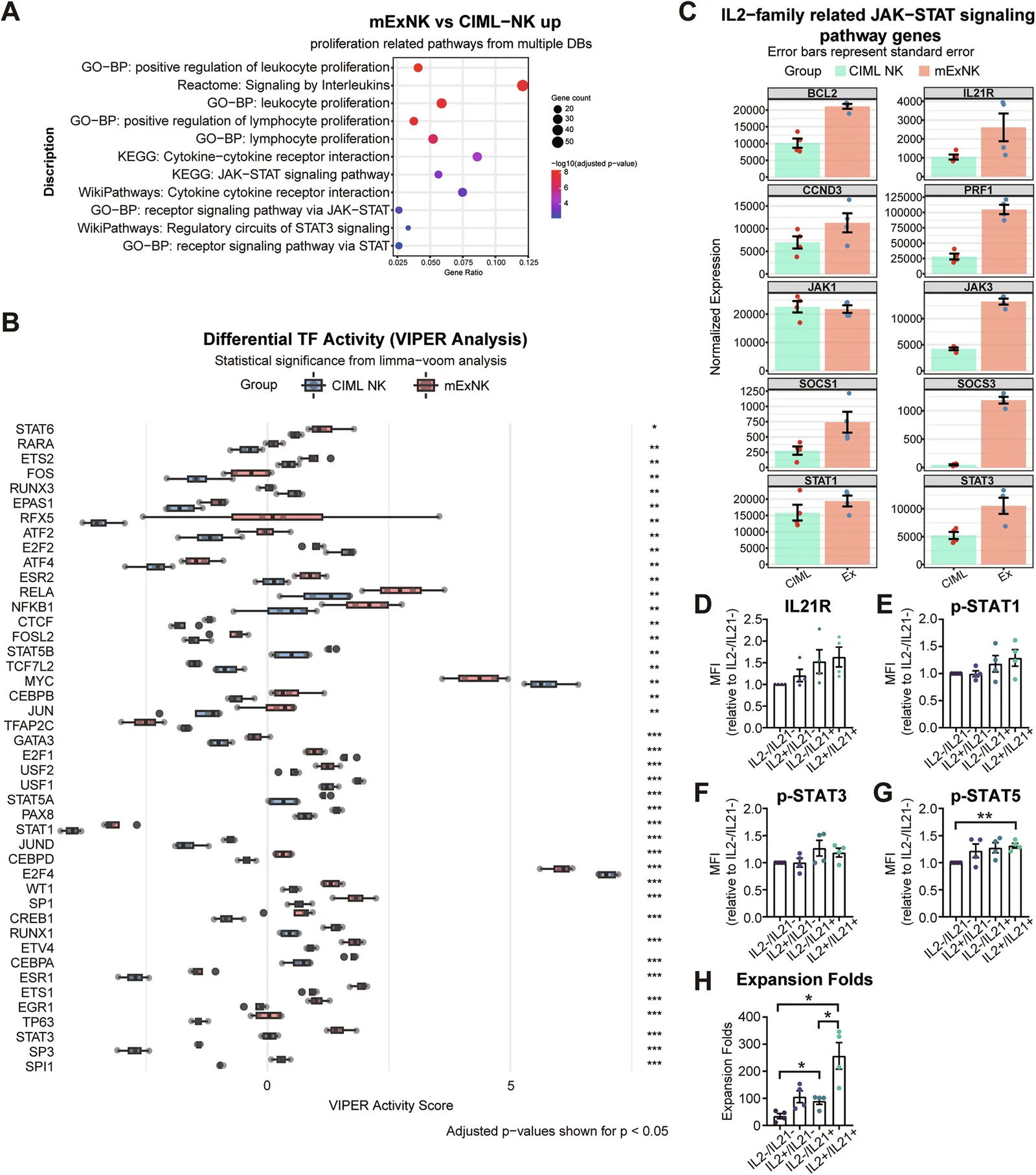
IL-2/15/21 synergistically induce JAK-STAT signaling pathway to promote NK cell expansion. **A** Over representation analysis (ORA) of the upregulated DEGs between mExNK vs CIML NK from 4 differential databases (KEGG, GO-BP, Reactome, Wikipathways). **B** Bar plots showing the differential transcriptional factors (TFs) activity from VIPER (Visualization Pipeline for RNA-seq analysis) analysis between mExNK and CIML NK groups. *p < 0.05, **p < 0.01, ***p < 0.001. **C** Bar plots showing the comparison of representative genes in JAK-STAT signaling pathway between mExNK and CIML NK groups. **D** Relative MFI of the cells expressing IL21R in IL2-/IL21-, IL2+/IL21-, IL2-/IL21+ and IL2+/IL21+ groups (*n* = 4). **E–G** The relative MFI of the cells expressing p-STAT1 (Tyr701), p-STAT3 (Tyr705) or p-STAT5 (Tyr694) in IL2-/IL21-, IL2+/IL21-, IL2-/IL21+ and IL2+/IL21+ groups (*n* = 4). **p < 0.01. **H** Expansion fold change in IL2-/IL21-, IL2+/IL21-, IL2-/IL21+ and IL2+/IL21+ groups (*n* = 4). *p < 0.05.

Over-representation analysis of genes upregulated in mExNK versus CIML NK cells revealed consistent and significant enrichment of the JAK-STAT signaling pathway across multiple databases (Fig. 6A). This aligns with the known roles of cytokines in our culture system: IL-15 supports NK cell survival [50], IL-21 activates *STAT3* and upregulates *MYC* to regulate proliferation and metabolism [51], and IL-2 potently drives NK cell expansion and cytotoxicity [52, 53]. Transcription factor activity analysis further indicated differential activation of *STAT1*, *STAT3*, *STAT5A*, *STAT5B*, *NFKB1*, and *STAT6* in mExNK cells (Fig. 6B), and key JAK-STAT pathway genes (including *BCL2*, *CCND3*, *IL21R*, *JAK1*, *JAK3*, and *STAT1/3*) were more highly expressed in mExNK than in CIML NK cells (Fig. 6C).

Functional validation using different cytokine combinations confirmed the critical role of JAK-STAT activation. While IL-21 increased IL-21R expression non-significantly (Fig. 6D), it clearly enhanced phosphorylation of STAT1 and STAT3 (Fig. 6E, F). Co-stimulation with IL-2 and IL-21 further elevated p-STAT5 levels, indicating synergistic JAK-STAT activation (Fig. 6G). Expansion folds were 17-, 52-, 45-, and 128-fold for the IL-2-/IL-21-, IL-2+/IL-21-, IL-2-/IL-21+ , and IL-2 +/IL-21+ groups, respectively (Fig. 6H).

Together, the synergistic activation of the JAK-STAT pathway by IL-2 and IL-21 is a key mechanism driving the high-efficiency expansion of memory-like NK cells.

## Discussion

Over the past two decades, immunotherapy with functionally improved T and NK cells has emerged as a novel pillar of cancer treatment [6, 54]. Memory NK cells, particularly CIML NK cells, are highly promising due to their superior effector function and in vivo persistence [17, 31]. However, a major translational hurdle is their inefficient ex vivo expansion. Existing methods, such as those employing feeder cell-derived particles or specialized cytokine fusion proteins, face challenges related to safety, reproducibility, or complex manufacturing [55, 56].

To overcome these limitations, we developed a novel, feeder-free platform using readily available reagents. Our protocol induces memory-like properties through initial stimulation with CD16a antibody, IL-15, IL-18, and the ADAM17 inhibitor INCB3619 (to preserve CD16a). This is followed by a robust expansion phase driven by CD137 agonist (urelumab), IL-2, IL-15, and IL-21, achieving over 200-fold expansion in two weeks.

Our data comprehensively demonstrate that mExNK cells possess classic memory-like attributes across multiple layers. The mExNK cells exhibited high CD25 expression similar to CIML NK cells (Figs. 2C, 4G), much higher than PBNK and conventional ExNK (Fig. 2C and Supplementary Fig. 6E, 6L). They also underwent significant metabolic remodeling, evidenced by increased nutrient transporter expression, enhanced glycolysis, and transcriptional upregulation of OXPHOS and mTORC1 signaling pathways, which collectively support their enhanced effector functions (Figs. 2D–H, 4D, E, Supplementary Fig. 5F and 6E, F). At the epigenetic level, mExNK cells showed much lower methylation in the *IFNG* CNS-1 region than CIML NK cells (Fig. 2I, J, Supplementary Fig. 1D, E, Supplementary Fig. 2A–E). Supporting this permissive epigenetic state, mExNK cells produced more IFN-γ upon stimulation (Fig. 3A, F), demonstrating a direct link between their epigenetic and functional enhancements. Concurrently, mExNK cells expressed higher levels of cytotoxic molecules, including Perforin and Granzyme B (Fig. 3A–G, Fig. 4F and Supplementary Fig. 6G–I), and exhibited superior in vitro and in vivo tumor-killing and persistence capacity (Fig. 3H–J, Fig. 5B–F and Supplementary Fig. 3A–I).

Foltz et al. originally identified CD117-CD57-NKG2A+ CD39+ enriched memory-like (eML) NK cells as the subpopulation possessing genuine immune memory [45]. Our mRNA-seq analysis revealed a high degree of similarity between the transcriptomes of mExNK cells and these eML NK cells, including concordant changes in key genes (Fig. 4H). Specifically, compared to CIML NK cells, mExNK cells expressed lower levels of KIT (encoding CD117) and B3GAT1 (encoding CD57), and higher levels of ENTPD1 (encoding CD39) (Fig. 4H, Supplementary Fig. 1A), suggesting a greater enrichment of the bona fide eML subset. Integrating these phenotypic, metabolic, epigenetic, transcriptomic, and functional analyses, we conclude that mExNK cells likely possess more robust immune memory characteristics than CIML NK cells.

Therefore, despite undergoing expansion, mExNK cells retain strong immune memory properties. The expansion of mExNK cells significantly enhances the production efficiency and clinical therapeutic potential of traditional CIML NK cells, while simultaneously improving their functionality, offering greater clinical applicability. However, how to further improve the expansion efficiency of memory-like NK cells remains a challenge to be overcome.

## Materials and methods

### Mice

Housing and all experimental protocols for mice used in this study were performed in accordance with the guidelines established by the Institutional Animal Care and Use Committee in the East China Normal University. NCG mice (NOD/ShiLtJGpt-Prkdc^em26Cd52^Il2rg^em26Cd22^/Gpt) were purchased from The GemPharmatech. If not stated differently, 6-8-week-old male mice were used for all experiments.

### Cell culture

#### Cell lines

The source of THP-1-Luciferase, K562 and Raji tumor cell lines were described in Table S3. They were all cultured in RPMI 1640 (Gibco) supplemented with 10% FBS (Gibco).

#### PBNK cells

Human peripheral blood NK (PBNK) cells were isolated from PBMCs using the NK Cell Isolation Kit (Miltenyi) according to the manufacturer’s instructions. Purity (CD56+&CD3-) was assessed, and only samples with >90% purity were used for subsequent experiments. Cells were cultured in HIPP maintenance medium, which was composed of HIPP-T009 medium (Bioengine) supplemented with 5% UltraGRO™-Advanced Serum Replacement (Helios) and 1 ng/mL IL-15.

#### CIML NK cells

Freshly purified PBNK were pre-activated with IL-12 (10 ng/mL), IL-15 (50 ng/mL), and IL-18 (50 ng/mL) for 24 h to generate cytokine induced memory-like NK cells (CIML NK), then washed, and allowed to rest for another 3 days in HIPP maintenance medium as described [16, 17, 57].

#### cExNK cells

Freshly purified PBNK cells were activated and expanded with NK cell Activation/Expansion Kit (Miltenyi) by using the NKp46/CD2 coupling microbeads as described per manufacturer’s instructions. The expansion medium was HIPP-T009 medium (Bioengine) supplemented with 5% UltraGRO™-Advanced Serum Replacement (Helios), 500 U/mL rhIL-2 (PeproTech). After 14 days, this conventional expanded NK cells (cExNK) were used in the FACS tests and ELISA assay.

### Isolation and expansion of mExNK cells

Healthy anonymous human PBMC were obtained by Ficoll centrifugation of cells from leukoreduction filters following platelet apheresis. The CD3-depleted PBMCs (with a residual T cell percentage ≤ 1%) were isolated using Dynabeads™ CD3 (Thermo) as described per manufacturer’s instructions.

Then dT-PBMCs were placed in anti-CD16a antibody (T&L) immobilization plate containing HIPP-T009 medium (Bioengine) supplemented with 5% UltraGRO™-Advanced Serum Replacement (Helios), 500 IU/mL rhIL-2 (PeproTech), 50 ng/mL rhIL-15 (T&L), 50 ng/mL rhIL-18 (T&L) and 4 µM ADAM inhibitor (MCE) at 37°C in a humidified atmosphere containing 5% CO2. After 2 days, fresh medium with 500 IU/mL rhIL-2 (PeproTech), 50 ng/mL rhIL-15 (T&L) and 1 μg/mL Urebumab (MCE) were added. From day 4, medium containing 500 IU/mL rhIL-2 (PeproTech), 50 ng/mL rhIL-15 (T&L) and 50 ng/mL rhIL-21 (T&L) was replenished as necessary during the NK expansion process.

### IL-2/21 expansion test

The treatment from day 0 to day 4 (including cell isolation, memory induction, and early expansion) was identical to the protocol used for generating mExNK cells. Beginning on day 4, cells from the same donor were divided into four culture groups: Group IL-2-/IL-21-: medium supplemented with 50 ng/mL rhIL-15 only; Group IL-2+ /IL-21-: medium containing 50 ng/mL rhIL-15 and 500 IU/mL rhIL-2; Group IL-2-/IL-21+ : medium containing 50 ng/mL rhIL-15 and 50 ng/mL rhIL-21; Group IL-2 + /IL-21+ : medium containing 50 ng/mL rhIL-15, 500 IU/mL rhIL-2, and 50 ng/mL rhIL-21.

All groups were cultured for an additional 10 days with fresh medium with associated cytokines added every other day, after which cell expansion was quantified, and the expression levels of IL-21R and phosphorylated STAT1, STAT3, and STAT5 (p-STAT1/3/5) were analyzed.

### Flow cytometry analysis

For analysis of surface markers, cells were stained in PBS (Gibco) containing 1% FBS (Gibco). Surface proteins were stained at 4°C for 30 min. Intracellular staining of SLC1A5, SLC7A5, GLUT1 and cytokines was performed with Cytofix Fixation Buffer (BD) and Perm/Wash Buffer I (BD), according to the manufacturer’s instructions. Intranuclear staining of p-STAT1/3/5 was performed with BD Phosflow™ Fix Buffer I and BD Phosflow™ Perm Buffer III, according to the manufacturer’s instructions. Data were obtained using a CytoFLEX flow cytometer (Beckman Coulter) and analyzed using FlowJo Software. All antibodies and their working conditions are listed in Table S1.

### Incucyte-based NK cytotoxicity assays

K562 target cells were labeled with Incucyte^®^ Cytolight Rapid Green Dye (Sartorius). Then labeled K562 cells were plated at a concentration of 1e4 per well in 96-well flat bottom plate. Expanded NK cell were added at E:T ratios ranging from 1:1 to 40:1. All co-cultures were performed in HIPP maintenance media. Target viability (green area) was imaged every 1 h for 1day and normalized to the target cell-only control group.

### Degranulation assay and cytokine production

PBNK, CIML NK and mExNK cells were co-cultured with K562 tumor cells for 6 h at an effector/target ratio of 1:1 in HIPP maintenance medium. For degranulation assays, anti-CD107a mAb was added at the start of the assay in 1:100 dilution ratio. To assay cytokine production, Brefeldin A and monensin (eBioscience) were added within the first hour of co-culture. After 5 h, cells were washed and stained for cell surface marker hCD56 (1:200 dilution), fixed/permeabilized and intracellularly stained for IFN-γ, TNF-α, Perforin, Granzyme B (1:100 dilution).

### ELISAs

PBNK, CIML NK and ExNK cells were co-cultured with K562 tumor cells at an effector/target ratio of 1:1 in HIPP maintenance medium. The supernatant was collected after 24 h, and Granzyme B, Perforin, IFN‑γ secretion was measured using the Granzyme B Valukine ELISA Kit (R&D Systems), ELISA Flex Human Perforin (HRP) (MABTECH) and Human IFN-γ Broad range ELISA Kit (STARTER, China) individually according to the manufacturer’s instructions.

### Metabolism assessment

PBNK, CIML NK and mExNK cells were plated at 1.5×10⁵ cells/well in Seahorse XF24 plates. For ECAR assays, Glucose, oligomycin and 2-deoxyglucose (2-DG) at a final concentration of 10 mM, 2.5 µm and 50 mM individually, were serially injected. For OCR assays, oligomycin, FCCP, antimycin A and rotenone at a final concentration of 2.5 µM, 1 µM, 2.5 µM and 2.5 µM, were serially injected. All reagents were listed in Table S2.

Glycolysis was defined as the ECAR after glucose addition minus basal ECAR. Glycolytic capacity was the ECAR after oligomycin injection minus basal ECAR. Glycolytic reserve was the maximum ECAR minus the ECAR after glucose. For mitochondrial function, basal respiration was the OCR prior to inhibitor addition. ATP respiration was calculated as basal OCR minus OCR after oligomycin. Spare respiratory capacity (SRC) was the maximum OCR minus basal OCR.

### Conventional bisulfite sequencing of IFNG-CNS1 region

Genomic DNA from PBNK, CIML NK and ExNK cells was extracted with FastPure Blood/Cell/Tissue/Bacteria DNA Isolation Mini Kit (Vazyme, DC112-01) and treated with EZ DNA Methylation-Direct Kit according to the manufacturer’s instructions. Bisulfite-treated DNA was subjected to PCR amplification and cloned into pGEM-T vector (Promega). Individual clones were sequenced by standard Sanger sequencing. Data were analyzed by BISMA (http://services.ibc.uni-stuttgart.de/BDPC/BISMA/) [58].

Primers sequences for the bisulfate PCR and sequencing were listed in Table S4.

### Repeated tumor rechallenge assays

Persistence of NK cells in vitro were evaluated in a serial tumor challenge assay as described before [39]. In brief, 2.5×10^4^ or 1.25×10^4^ NK cells and 2.5×10^4^ target cells (K562, Raji or THP-1) labeled with cytolight red (Sartorius) were co-cultured in a poly-D-lysine pre-coated 96-well plate using HIPP maintenance medium. Maintained the plate in a 37°C, 5% CO_2_ incubator for 6 days to perform 3 repeated tumor rechallenge, additional cytolight red labeled tumor targets in fresh medium administered to the co-culture every other day. Target viability (red area) was imaged every 2 h for over 140 h using an IncuCyte S3 live cell imaging and analysis system and normalized to initial values (0d0h0m).

### In vivo tumor xenograft models

For in vivo experiments testing ExNK cell function again THP-1 tumor cells in a single infusion model, 6- to 8-week-old male NSG mice were injected intravenously in tail-vein with 2e6 luciferase expressing THP-1 tumor cells. There days later mice were distributed into 2 groups randomly, each group with at least 8 mice. mExNKs were resuspended in 1e7/0.2 mL PBS buffer and injected intravenously in tail-vein of tumor bearing mice. Transferred NK cells were supported with rhIL-2 (50000U/mice) and rhIL-15 (10 ng/mice) by intraperitoneal (i.p.) injection at least 2 times every week for up to 3 weeks. BLI was performed weekly to monitor tumor progression, at the same time monitor the mice body weight weekly. THP-1-Luciferase tumor-bearing mice without NK cell transfer (PBS) served as controls. Animal technicians were blinded to expected outcomes. The experiment was approved by the Institutional Animal Care and Use Committee in the East China Normal University.

BLI was conducted using an IVIS Spectrum, and images were analyzed using Live Imaging Software (Perkin Elmer). Survival was graphed as Kaplan-Meier curves, and the log-rank (Mantel-Cox) test was used to determine statistical significance.

### Persistence of mExNK cells in NSG mice

After 1 week of acclimation, a single dose of 1×10^7^ mExNK cells was injected intravenously through the tail vein into male NSG mice with rhIL-2 (50000U/mice) and rhIL-15 (10 ng/mice) (i.p. injection, 2 times per week for 3 weeks) support. For blood collection, about 100 μl blood obtained from orbital venous bleeds using EDTA anticoagulant at 1, 2, 4 weeks post-infusion for immediate flow cytometry.

Blood sample were incubated with the appropriate antibody fluorophore conjugates (mouse CD16/32 Fc block (Biolegend, 1:100), viability Live/Dead (Thermo, 1:1000), PE-hCD45 and APC-hCD56 (Biolegend, 1:50)) for 30 mins on ice in staining buffer (DPBS + 2%FBS + 2 mM EDTA). Then stained blood samples were incubated with ACK buffer (Gibco) for 5 min on ice to lyse red blood cells. After lysis, samples were washed with cold staining buffer once, then resuspended in 90 μl staining buffer plus 10 μl CountBright absolute counting beads (Thermo), and data was acquired in CytoFLEX flow cytometer (Beckman Coulter). Population densities were quantified to hCD45+&hCD56+ cells/total cells using in sample CountBright beads.

### HLA-E overexpression assay

The human *HLA-E* gene was cloned into the lentiviral vector pLV-CMV-EGFP-PGK-Puro; the empty vector was used as a control. Lentivirus packaging was performed by Shanghai LifeSpan Biotechnology Co., Ltd. K562 cells were transduced with the lentivirus at a multiplicity of infection (MOI) of 30. Following transduction, cells were selected with 2 ng/mL puromycin for 6 days. Prior to the killing assay, both GFP expression and HLA-E surface expression were confirmed by flow cytometry.

### In vitro safety assessment

To assess CRS potential, we established co-cultures containing effector cells, K562 cells, and monocytes. T effector cells served as controls. To avoid alloreactivity, monocytes and T cell-derived PBMCs came from the same donor, while NK cells and monocytes came from different donors (donor details in Tables S5–S6).

Fresh monocytes were isolated using the Pan Monocyte Isolation Kit. Effector cells (2×10⁵), monocytes (1×10⁵), and K562 cells (1×10⁵) were seeded in 200 µL medium per well in 96-well plates. After 24 h, supernatants were collected, diluted 1:3, and assayed for IL-6, IFN-γ, and TNF-α using a Human Th1/Th2 Cytokine Kit.

For GvHD assessment, we measured cytotoxicity and cytokine secretion of mExNK cells against allogeneic PHA-blasts, autologous PHA-blasts, or K562 cells. Controls included CD3/CD28-activated T cells, CIML NK cells, and PBNK cells (donor details in Tables S5 and S7).

PHA-blasts were generated by treating PBMCs with 10 µg/mL PHA for 5-7 days, followed by 20 ng/mL mitomycin C for 30 min to block proliferation. After washing, PHA-blasts and K562 cells were labeled with CytoRed dye and co-cultured with effector cells at an E:T ratio of 1:1 for 48-h cytotoxicity and 6-h cytokine tests.

### RNA-seq sample preparation, sequencing, and data analysis

Total RNA was extracted using TRIzol Reagent (Thermo). Sequencing libraries were constructed with the VAHTS Universal V10 RNA-seq Library Prep Kit for MGI (Vazyme), size-selected for 200–300 bp cDNA fragments, amplified with Phusion DNA polymerase (NEB), and paired-end sequenced on a DNBSEQ-T7 platform (Shanghai BIOZERON).

For data analysis, raw reads were trimmed and quality-controlled with Trimmomatic. Clean reads were aligned to the human reference genome (hg38) using HISAT2, and data quality was assessed with Qualimap. Ribosomal RNA content was evaluated by aligning to the rRNA database using Bowtie2. Gene expression levels were quantified as FPKM using featureCounts. Differential expression analysis was performed with the edgeR package in R, with an adjusted *p*-value < 0.05 defining significance.

### Statistical analysis

Statistical details of the experiments are provided in the figures and corresponding legends. Unless otherwise stated, all experiments were performed with a minimum of three independent biological replicates per experimental group (*n* = 3). No specific inclusion or exclusion criteria were pre‑established.

Figures were prepared using GraphPad Prism8. All data are presented as mean ± s.e.m, excepting informed specially. Statistical significance excepting the survival curve of mice and the ELISA result of IFN-γ was calculated using an unpaired, two-tailed student’s t-test with 95% confident intervals. ns, not significant (*p* > 0.05). *p < 0.05, **p < 0.01, ***p < 0.001. The statistical significance of mice survival curve was calculated using Log-rank (Mantel-Cox) test. The statistical significance of IFN-γ ELISA result was calculated using F-test.

## Supplementary information

Supplementary materials

## Data Availability

mRNA-sequencing data is available via GEO under accession numbers GSE311121. Additional data will be provided upon reasonable request to the lead contact, Prof. Zhiying He (zyhe@tongji.edu.cn).
