# Supplementary material for: Cytokine-induced expandable memory NK cells with significant metabolic, epigenetic remodeling, and persistence properties
Source: Cell Death Discov. 2026 Jul 21;12:417. doi: 10.1038/s41420-026-03260-2 (PMC13672343; doi:10.1038/s41420-026-03260-2)
Supplement: Supplementary file 1 [file 41420_2026_3260_MOESM1_ESM.pdf]

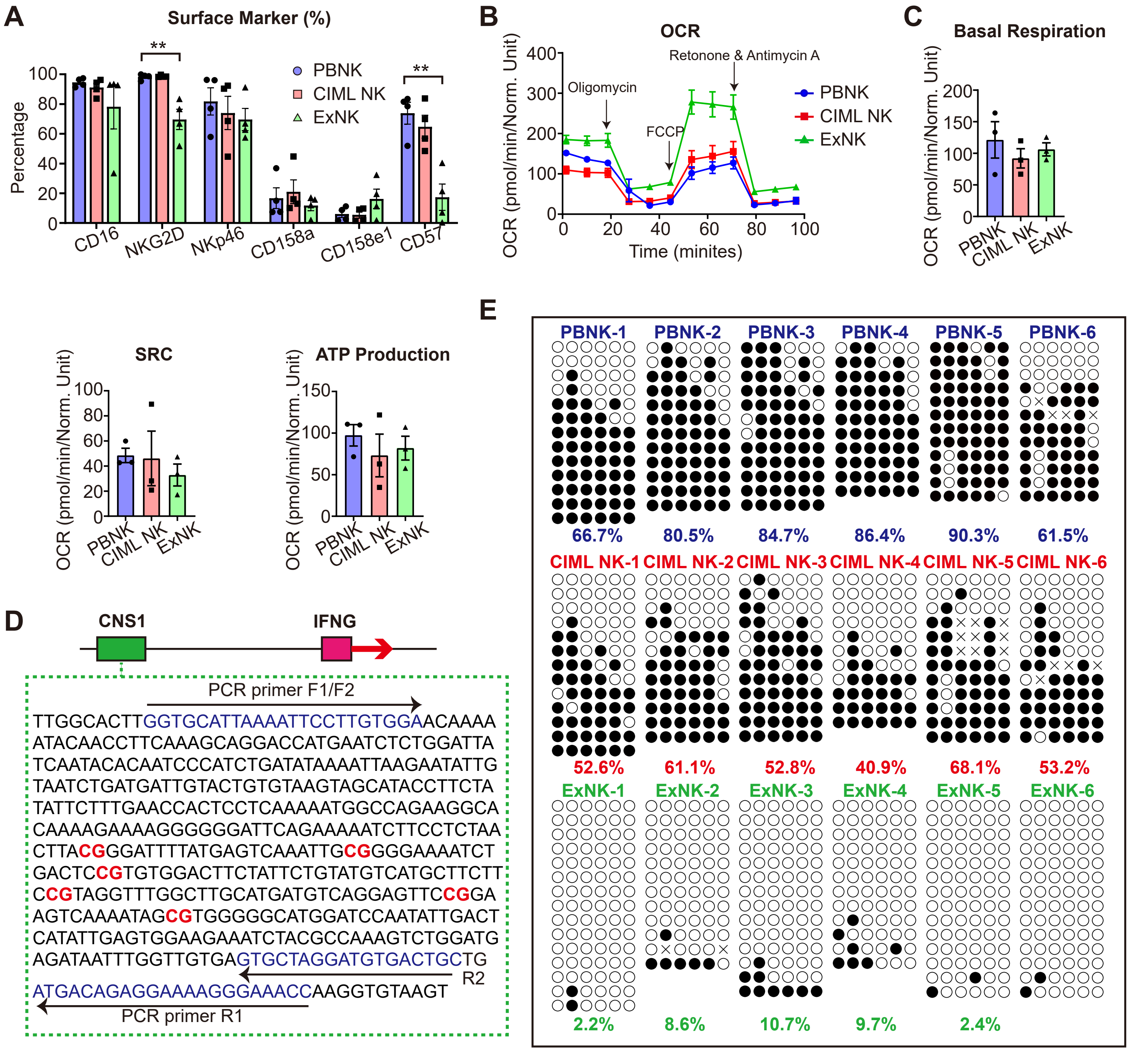

Fig S1. The phenotype, metabolism and epigenetic remodeling of the memory-like expanded NK cells (mExNK).
A. The positive percentage of surface marker expression (CD16, NKG2D, NKp46, CD158a, CD158e1 and CD57) in PBNK, CIML NK and ExNK cells (n=4). ** $p < 0.01$. **B.** Seahorse cell assay showing oxygen consumption rate (OCR) profile in PBNK, CIML NK and ExNK cells, respectively (n=3). All samples were normalized to cell number. Each time point represented the mean of three replicates from one sample and the SEM is represented by the error bars. **C.** Quantification of OCR in basal respiration, spare respiratory capacity (SRC) and ATP production (n=3). Three independent experiments performed in triplicate. **D.** Scheme of six CpG sites evaluated for DNA methylation in the CNS-1 region of IFNG. We have designed a two-rounds PCR to amplify the CNS-1 DNA. The first round PCR using the primers F1 and R1, the second round PCR using the primers F2 and R2. The nucleotides of F1 and F2 were identical (Table S4). **E.** Bisulfite sequencing results of CNS-1 region of IFNG gene in PBNK, CIML NK and ExNK samples (n=6). White and black circles indicated hypomethylated and hypermethylated CpG sites, respectively.

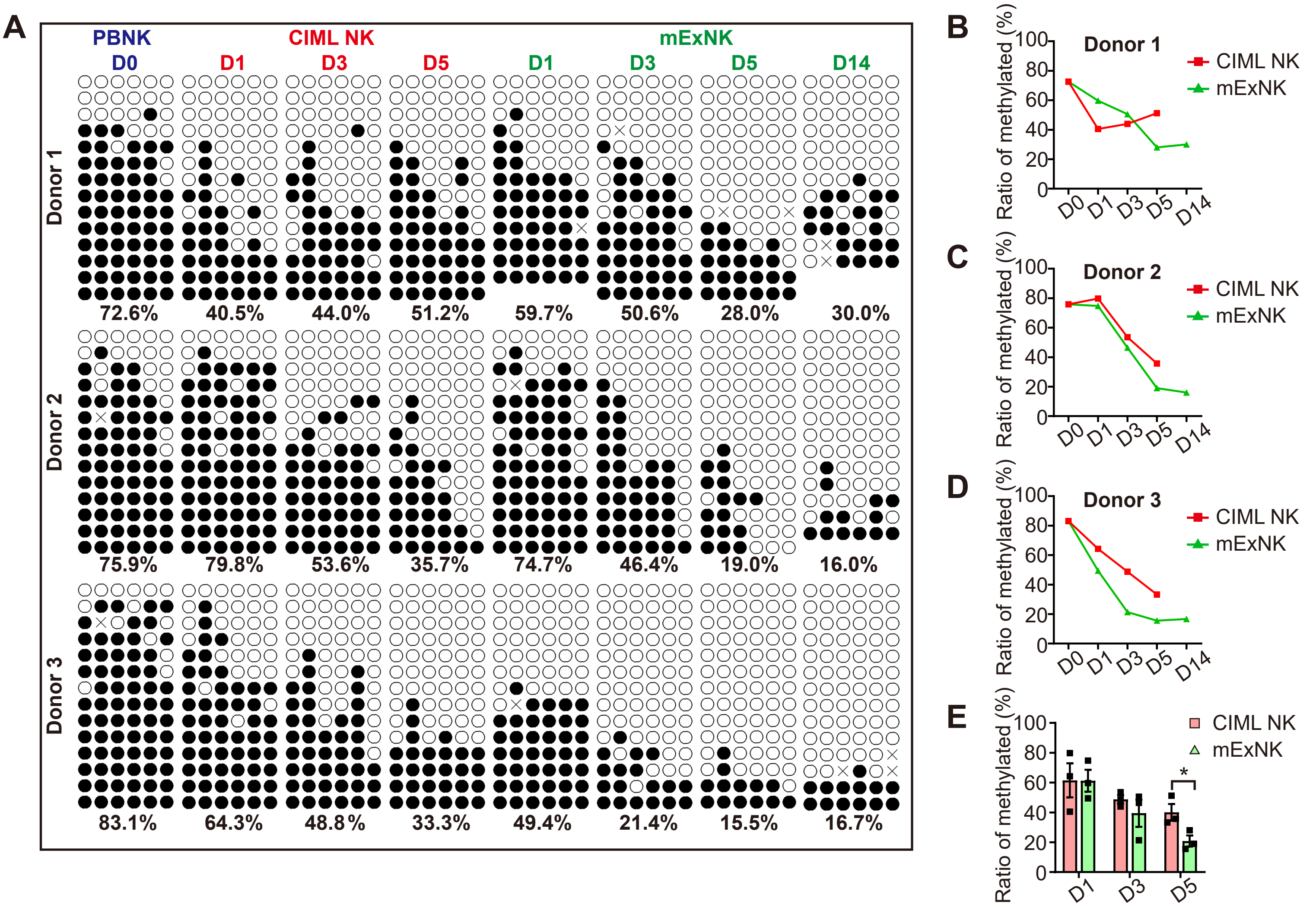

Fig S2. Effects of different treatment durations on epigenetic reprogramming of CIML NK and mExNK cells. **A.** Bisulfite sequencing results of CNS-1 region of *IFNG* gene in PBNK, CIML NK and mExNK samples from three donors at different time point (n=3). **B-D.** DNA methylation levels of the CNS-1 region in donor 1, donor 2, and donor 3, respectively. **E.** Average DNA methylation level of CIML NK and mExNK in d1, d3 and d5. * $p < 0.05$.

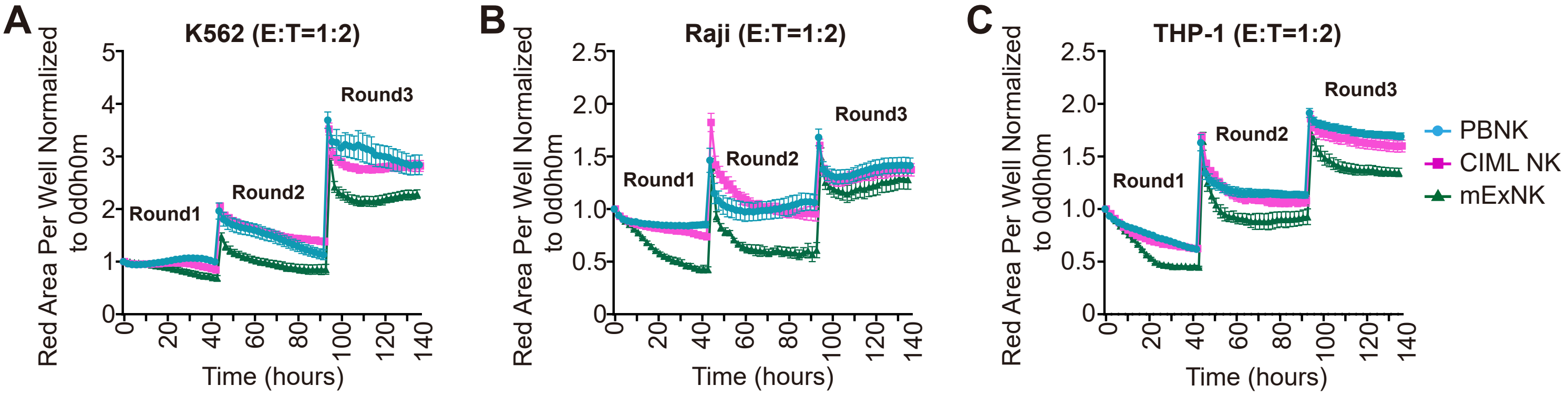

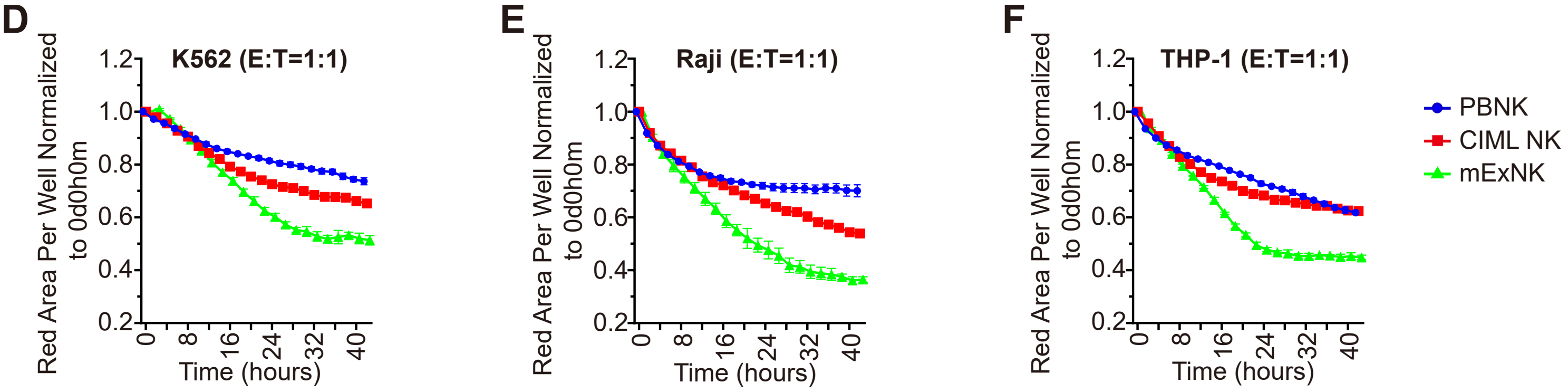

D

	E vs C	E vs P	C vs P
6h	ns	ns	ns
18h	***	***	***
36h	***	***	***

E

	E vs C	E vs P	C vs P
6h	*	*	ns
18h	***	***	*
36h	***	***	**

F

	E vs C	E vs P	C vs P
6h	**	**	ns
18h	***	***	***
36h	***	***	*

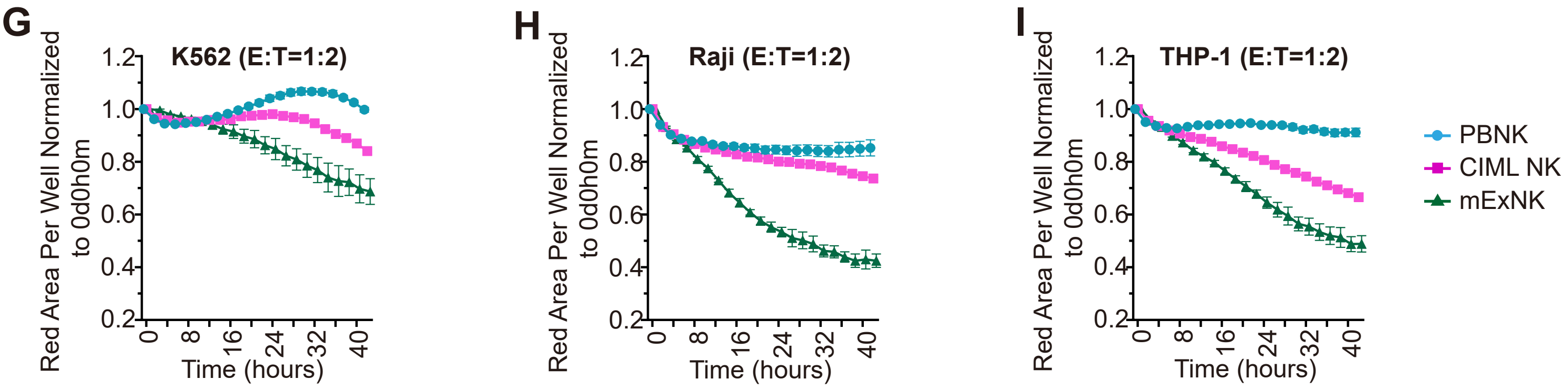

G

	E vs C	E vs P	C vs P
6h	ns	ns	ns
18h	**	**	*
36h	**	***	***

H

	E vs C	E vs P	C vs P
6h	**	***	ns
18h	***	***	**
36h	***	***	*

I

	E vs C	E vs P	C vs P
6h	ns	**	*
18h	***	***	***
36h	***	***	***

Fig S3. Assessment of the in vitro cytotoxicity activities and persistence ability of mExNK cells. A-C. Data were generated with Incucyte. Cumulative data from three repeated-round tumor rechallenge assay showing K562 (A), Raji (B), THP-1 (C) viability, quantified by relative red intensity, in each coculture condition with 1:2 E:T ratio over the course of 140 hours (n=3). Data are shown as mean ± SEM of 3 replicates per timepoint. **D-I:** Statistic of the target cell viability, quantified by relative red intensity at 6h, 18h, 36h (n=3). PBNK, CIML NK and mExNK cells were used as effectors at the indicated E:T ratios (E:T=1:1 (D, E, F) and E:T=1:2 (G, H, I)) in the first 40-hour IncuCyte-based tumor rechallenge assays with K562 (D and G), Raji (E and H), THP-1 (F and I) target cells. ns, no significant, * $p < 0.05$, ** $p < 0.01$, *** $p < 0.001$.

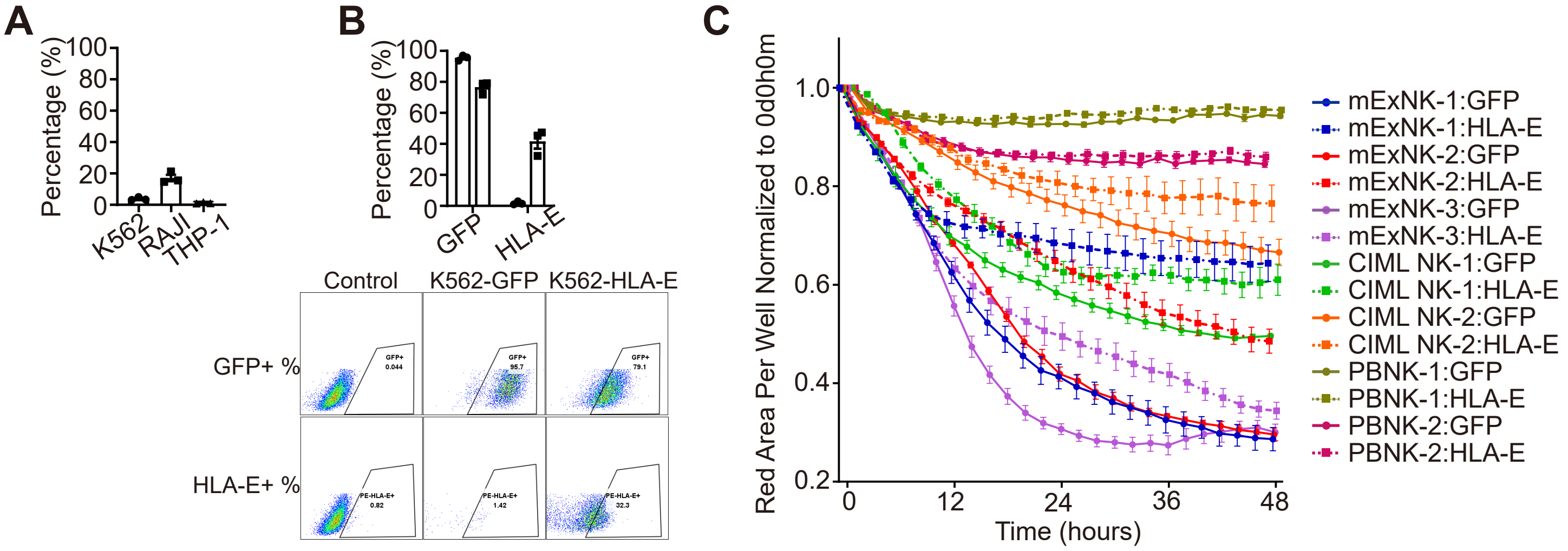

Fig S4. High NKG2A expression inhibits killing of HLA-E+ tumor cells. A. HLA-E expression on K562, Raji, and THP-1 cells (n=3). **B.** GFP positivity and HLA-E expression on control and HLA-E-overexpressing K562 cells (n = 3). **C.** Incucyte® analysis of NK cell-mediated killing of control vs. HLA-E-overexpressing K562 cells over 48 hours (E:T=1:1). mExNK cells were from three batches; CIML NK and PBNK cells were from two batches.

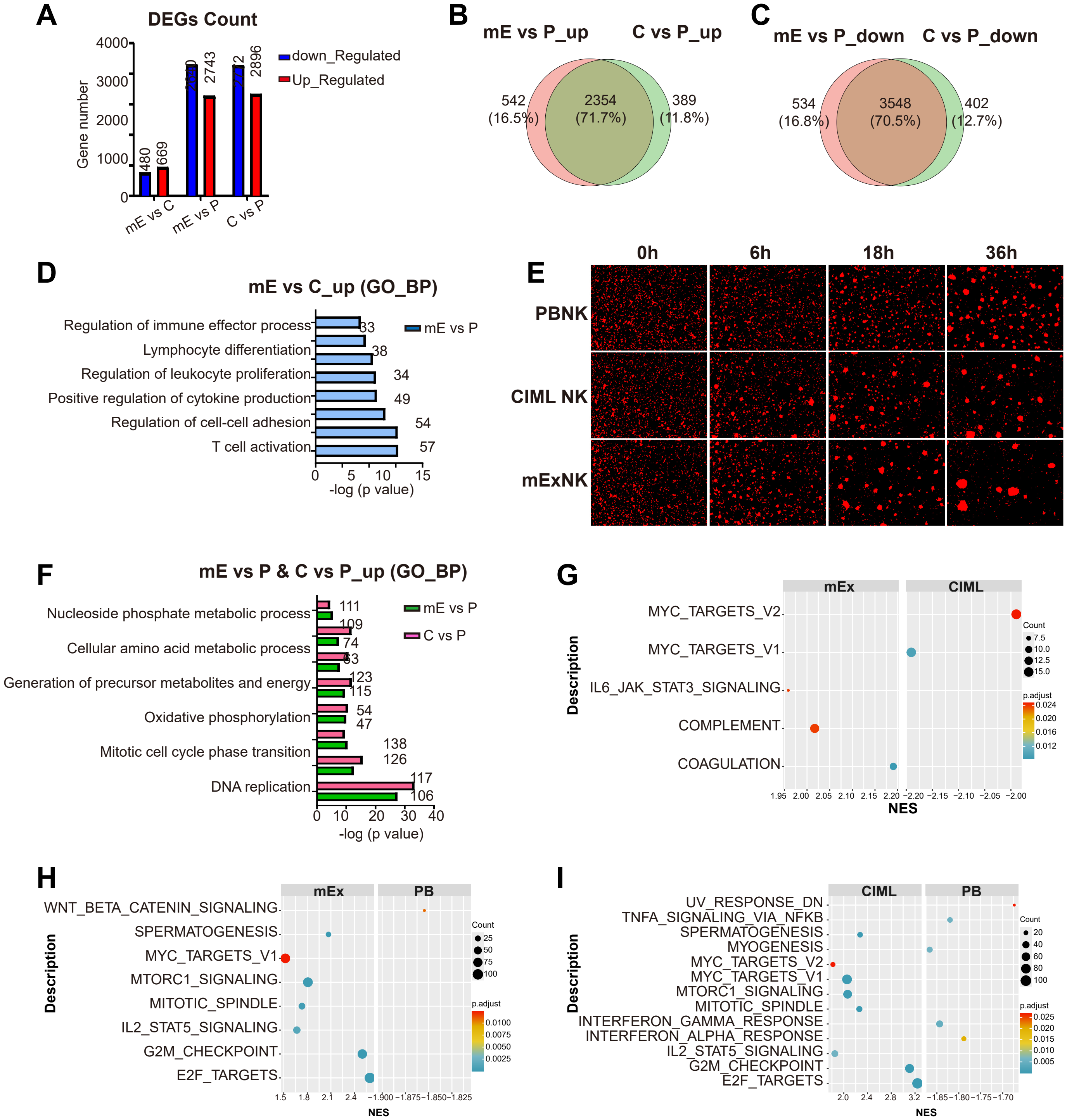

Fig S5. Analysis of mRNA sequencing. A. Average number of DEGs between mExNK and CIML NK group, mExNK and PBNK group, or CIML NK and PBNK group. **B.** Venn diagram of the up-regulated genes between mExNK vs PBNK and CIML NK vs PBNK. **C.** Venn diagram of the down-regulated genes between mExNK vs PBNK and CIML NK vs PBNK. **D.** Gene ontology analysis of the upregulated DEGs between groups. mExNK vs CIML NK was indicated with blue bar. Numbers within bars indicated the number of genes identified within the corresponding gene ontology categories. **E.** Fluorescence microscopy (20× magnification) of cultures at 0, 6, 18, 36 hours post-incubation of PBNK, CIML NK or mExNK cells with K562 tumor cells at an E:T of 1:1 from the tumor rechallenge assay in Fig 3I. Red indicated Cytolight Red staining of K562 tumor cells; **F.** Gene ontology analysis of the upregulated DEGs between groups. mExNK vs PBNK was indicated with green bar, CIML NK vs PBNK was indicated with pink bar. Numbers within bars indicated the number of genes identified within the corresponding gene ontology categories. **G-I.** GESA analysis performed using MSigDB Hallmark protein-protein interaction network. Only significantly enriched pathways ($p < 0.05$) were displayed. Dot size corresponds to the number of genes in the pathway. Dot color corresponds to the significance of the pathway.

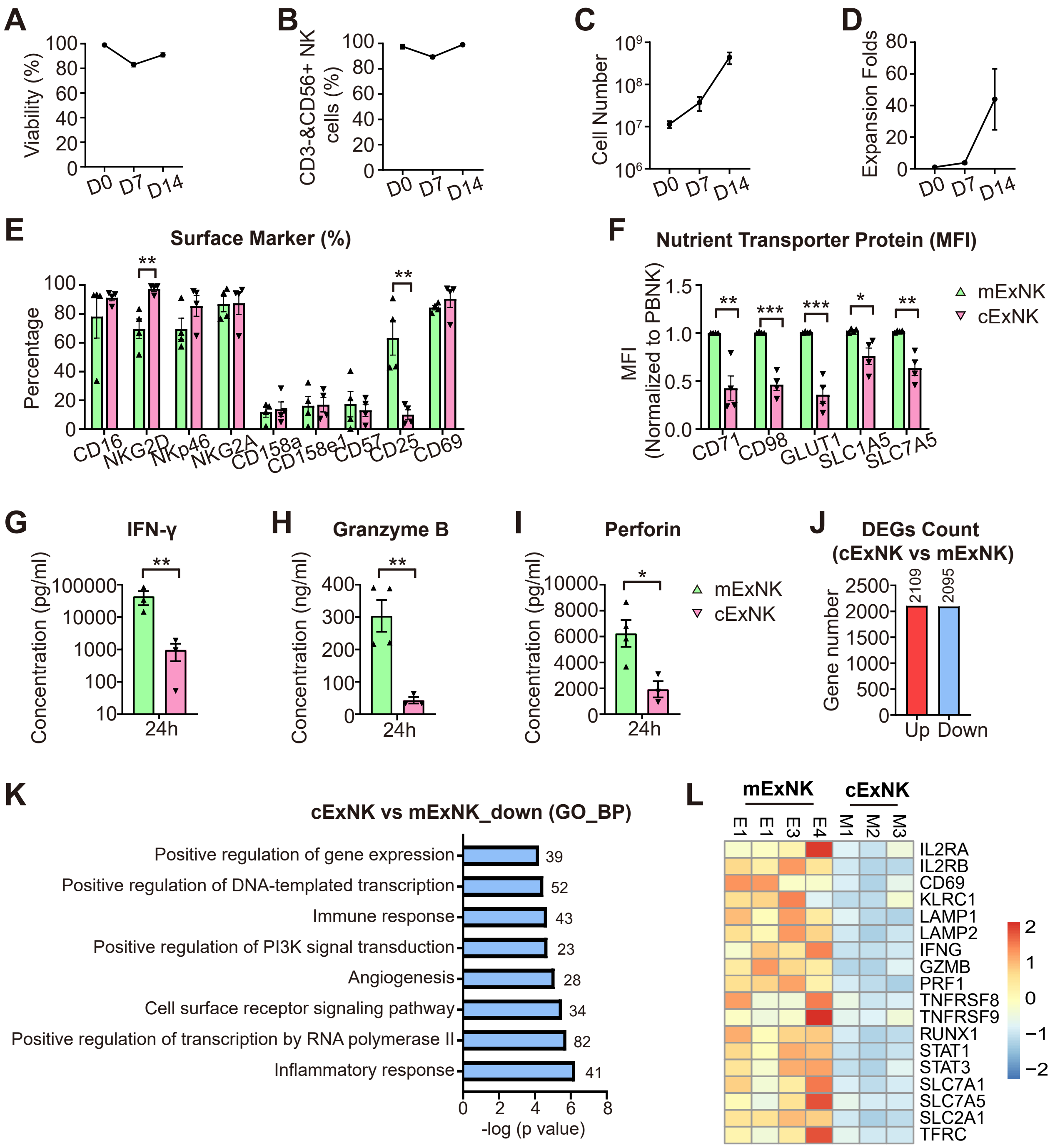

Fig S6. The characteristics of conventional expanded NK cells (cExNK). A. The viability of human cExNK cells during expansion for 14 days. Cells were collected weekly and analyzed by AOPI staining (n=4). **B.** The purity of NK cells identified by the flow cytometry of CD3-&CD56+ during expansion period (n=4). **C.** Increasing of total cell number over expansion period (n=4). **D.** Expansion fold change of cExNK cells (n=4). **E.** The percentage of cExNK and mExNK cells that expressed CD16, NKG2D, NKp46, NKG2A, CD158a, CD158e1, CD57, CD25, CD69 from the flow cytometry assay (n=4). ** $p < 0.01$. **F.** The MFI of the cells expressing nutrient transport proteins (CD71, SLC3A2, SLC2A1, SLC1A5 and SLC7A5) in cExNK and mExNK samples measured by flow cytometry (n=4). * $p < 0.05$, ** $p < 0.01$, *** $p < 0.001$. **G-I.** The secretion of IFN-γ (G), Granzyme B (H), Perforin (I) was assessed by ELISA of the culture supernatants by co-culture of cExNK or mExNK cells with target K562 cells for 24 hours (n=3-4). **J.** Average number of DEGs between cExNK and mExNK group. **K.** Gene ontology analysis of the downregulated DEGs between cExNK and mExNK groups. cExNK vs mExNK was indicated with blue bar. Numbers within bars indicated the number of genes identified within the corresponding gene ontology categories. **L.** Heatmap of partial genes in cExNK and mExNK samples. Horizontal axis indicated analysis of mExNK (n=4) and cExNK (n=3) groups. Vertical axis showed analysis of selected genes.

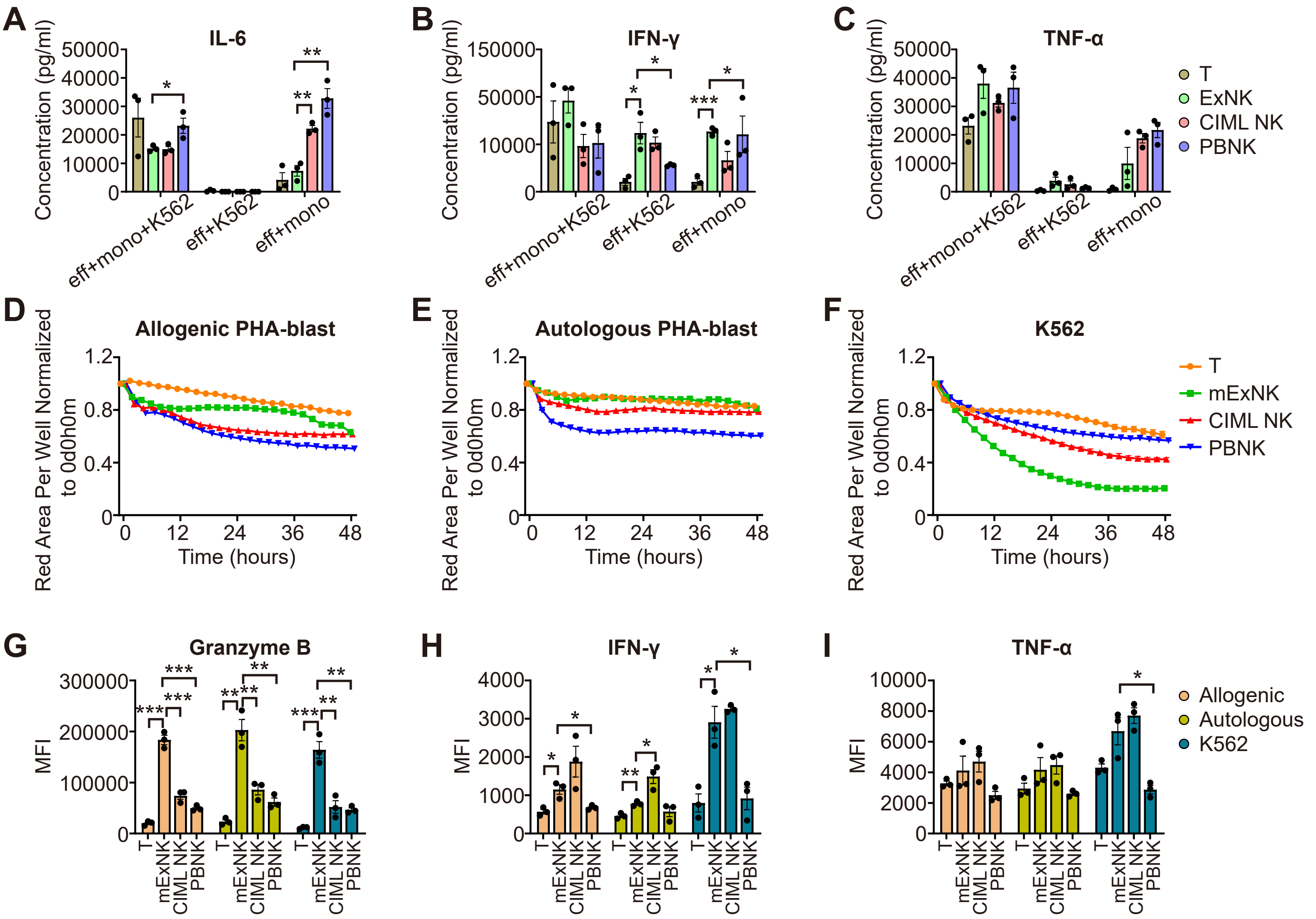

Fig S7. mExNK cells exhibit favorable *in vitro* safety profiles. A-C. Cytokine concentrations of IL-6 (A), IFN-γ (B), and TNF-α (C) in the supernatant after 24 hours of co-culture among effector cells, monocytes, or K562 tumor cells. "eff + mono + K562" indicates co-culture of effector cells (T cells, mExNK, CIML NK, or PBNK) with monocytes and K562 cells; "eff + K562" indicates co-culture of effector cells with K562 cells alone; "eff + mono" indicates co-culture of effector cells with monocytes alone. Three independent experiments were performed. * $p < 0.05$, ** $p < 0.01$. **D-F.** Killing analysis of allogeneic PHA-blasts (D), autologous PHA-blasts (E), or K562 cells (F) by different effector cells over 48 hours, monitored by the Incucyte® system (n=3). E:T=1:1. **G-I.** Expression of Granzyme B (G), IFN-γ (H), and TNF-α (I) after 6 hours of co-culture between effector cells (T cells, mExNK, CIML NK, and PBNK) and target cells (allogeneic PHA-blasts, autologous PHA-blasts, or K562 cells) (n=3). * $p < 0.05$, ** $p < 0.01$, *** $p < 0.001$.

Online Supplemental Tables

Table S1: Antibodies and reagents

Antibodies	Companies	Catalog Numbers	Working Concentration
APC anti-human IFN-γ Antibody	Biolegend	502512	1:100
PE anti-human TNF-α Antibody	Biolegend	502909	1:100
PE anti-human CD107a (LAMP-1) Antibody	Biolegend	328608	1:100
FITC anti-human Perforin	Biolegend	308104	1:100
APC anti-human/mouse Granzyme B Recombinant Antibody	Biolegend	372204	1:100
PE anti-human CD56 (NCAM) Antibody	Biolegend	362508	1:200
FITC anti-human CD56 (NCAM) Antibody	Biolegend	362546	1:200
APC anti-human CD56 (NCAM) Antibody	Biolegend	362504	1:200
PE anti-human CD45 Antibody	Biolegend	304008	1:200
PE anti-human CD3 Antibody	Biolegend	300408	1:200
PE anti-human CD25 Antibody	Biolegend	302606	1:200
PE anti-human CD69 Antibody	Biolegend	310906	1:200
PE anti-human CD314 (NKG2D) Antibody	Biolegend	320806	1:200
APC anti-human CD159a (NKG2A) Antibody	Biolegend	375108	1:200
PE anti-human CD57 Antibody	Biolegend	359612	1:200
PE anti-human CD335 (NKp46) Antibody	Biolegend	331908	1:200
APC anti-human CD337 (NKp30) Antibody	Biolegend	325210	1:200

PE anti-human CD336 (NKp44) Antibody	Biolegend	325108	1:200
APC anti-human CD16 Antibody	Biolegend	302012	1:200
PE anti-human CD158 (KIR2DL1/S1/S3/S5) Antibody	Biolegend	374904	1:200
FITC anti-human CD158b/j (KIR2DL2/L3/S2) Antibody	Biolegend	312604	1:200
FITC anti-human CD158e1 (KIR3DL1) Antibody	Biolegend	312706	1:200
APC anti-human CD71 Antibody	Biolegend	334108	1:50
FITC Mouse IgG1, κ Isotype Ctrl (FC) Antibody		400110	1:200
	Biolegend		
PE Mouse IgM, κ Isotype Ctrl Antibody		401609	1:200
	Biolegend		
PE Mouse IgG1, κ Isotype Ctrl Antibody	Biolegend	400112	1:200
FITC Mouse IgG2b, κ Isotype Ctrl Antibody	Biolegend	400310	1:100
APC Mouse IgG1, κ Isotype Ctrl Antibody	Biolegend	400120	1:200
Alexa Fluor™ 488 Rabbit Anti-Human GLUT1	BD	569321	1:50
Alexa Fluor™ 488 Rabbit IgG Isotype Control	BD	569346	1:50
PE Mouse Anti-Human CD98	BD	556077	1:50
PE Mouse IgG1, κ Isotype Control	BD	555749	1:50
Anti-SLC7A5/LAT1 antibody [EPR26260-66]	Abcam	ab305251	1:50
Anti-SLC1A5/ASCT2 antibody [CAL33]	Abcam	ab251591	1:50
Goat Anti-Rabbit IgG H&L / FITC secondary antibody	HZbscience	HZ-0295G	1:200

PE anti-human CD360 (IL-21R) Antibody	Biolegend	359506	1:50
Phospho-Stat1 (Tyr701) (D4A7) Rabbit mAb	CST	7649S	1:50
Phospho-Stat3 (Tyr705) (D3A7) XP® Rabbit mAb	CST	9145S	1:50
Phospho-Stat5 (Tyr694) (D47E7) XP® Rabbit mAb	CST	4322S	1:50

Table S2: Reagents

**Reagents**	**Companies**	**Catalog Numbers**
DH5α Competent Cells	Vazyme	C502-03
FastPure Blood/Cell/Tissue/Bacteria DNA Isolation Mini Kit	Vazyme	DC112-01
Taq™ Hot Start Version	TAKARA	R007A
pGEM®-T Easy Vector	Promega	A1360
EZ DNA methylation-gold kit	ZYMO research	D5005
Sodium pyruvate	Merck	P4562
D-(+)-glucose	Merck	G7021
2-Deoxy-D-glucose	Titan	01027516
Oligomycin	MCE	HY-N6782
FCCP	MCE	HY-100410
Rotenone	MCE	HY-B1756
Antimycin A	MK Bio	MS0070
XFe24 FluxPak mini	Agilent	102342-100
XF RPMI Base Medium	Agilent	103576-100

Poly-D-Lysine	Thermo	A3890401
TRIzol™	Thermo	15596026CN
Sodium acetate (3 M)	Thermo	AM9740
Glycogen (20 mg/mL)	Titan	G8234-500μL
Incucyte® Cytolight Red Reagent	Sartorius	4706
Incucyte® Cytolight Rapid Green Dye	Sartorius	4705
INCB3619 (ADAM inhibitor)	MCE	HY-12636
Dynabeads CD3	Thermo	11151D
HIPP-T009 Lymphocyte Serum-Free Medium	Bioengine	RG0101302
RPMI 1640	Gibco	C22400500BT
NK MACS® Medium, human	Miltenyi	130-114-429
FBS	Gibco	A5256701
UltraGRO	Helios	HPCPLCRL05
GlutaMAX	Thermo	35050061
BD Cytofix Fixation Buffer	BD	554655
BD Perm/Wash Buffer I	BD	557885
TruStain FcX™ PLUS (anti-mouse CD16/32) Antibody	Biolegend	156604
ACK lysis buffer	Gibco	A1049201
Human IFN-γ Broad range ELISA Kit	STARTER	S0C3025
ELISA Flex: Human Perforin (HRP)	MABTECH	3465-1H-6

Human Granzyme B Valukine ELISA Kit	R&D Systems	VAL142
Protein Transport Inhibitor Cocktail (500X)	Thermo	00-4980-03
NK Cell Isolation Kit, human	Miltenyi	130-092-657
NK cell Activation/Expansion Kit	Miltenyi	130-094-483
Recombinant Human IL-2 Protein	PeproTech	200-02-100
Recombinant Human IL-18 Protein	T&L Biotechnology	GMP-TL902
Recombinant Human IL-15 Protein	T&L Biotechnology	GMP-TL202
Recombinant Human IL-21 Protein	T&L Biotechnology	GMP-TL509
Recombinant Human IL-12 Protein	T&L Biotechnology	GMP-TL-508
Anti-human CD16 mAb	T&L Biotechnology	TL201
Urelumab	MCE	HY-P99055
Human Th1/Th2 Cytokine Kit	Starter	S0Q1001
Pan Monocyte Isolation Kit, human	Miltenyi	130-096-537
HLA-E overexpression lentivirus	LifeSpan	LS-Lenti-002-2

Table S3: Cell lines and mouse model

**Cell lines and mouse model**	**Source**	**Identifier**
K562 cell line	Cell Bank of the Committee on Type Culture Collection, Chinese Academy of Sciences	SCSP-5054
Raji cell line	Cell Bank of the Committee on Type	TCH44

	Culture Collection, Chinese Academy of Sciences	
THP-1 LUC+ cell line	Immocell	IML-066
NCG immunodeficient mice	GemPharmatech	T001475

Table S4: Primers

**Primers**	**Source**	**Sequence (5' to 3')**
CNS1-bisulfite-F1/F2	Sangon Biotech	GGTGTATTAAAATTTTTTGTGGA
CNS1-bisulfite-R1	Sangon Biotech	AATTTCCCTTTTCCTCTATCAT
CNS1-bisulfite-R2	Sangon Biotech	ACAATCACATCCTAACACTCAC

Table S5: HLA type of donors in the *in vitro* safety assessment

**Donor**	**Batch number**	**HLA-A***	**HLA-B***	**HLA-C***	**HLA-DQB1***	**HLA-DRB1***	**HLA-DPB1***
**1**	059K227	30:01,33:03	13:02,44:03	06:02,14:03	02:02,06:04	07:01,13:02	04:01,17:01
**2**	097K016	11:01,24:02	40:84,55:02	03:04,07:02	03:03,03:03	09:01,09:01	05:01:01G,13:01:01G
**3**	W054K176	03:01,24:02	35:02,35:03	04:01,04:01	02:02,03:01	07:01,11:04	03:01,104:01
**4**	WZ26042001	01:01,32:01	27:02,39:24	02:.02,07:01	05:03,06-03	14:04,16:02	05:01,21:01
**5**	097K017	02:01,02:07	15:11,46:01	01:03,03:03	03:03,03:03	09:01,09:01	02:01,05:01
**6**	097K018	02:11,11:01	40:01,40:06	03:04,15:02	03:01,15:01	02:01,09:01	02:01,06:01
**7**	W054K177	02:11,11:01	40:01,40:06	03:04,15:02	03:01,15:01	02:01,09:01	02:01,06:01
**8**	W054K193	23:01,34:02	15:03,35:02	02:10,04:01	05:02,06:04	11:01,13:02	04:01,04:01

**9**	W054K184	03:01,23:01	44:03,51:01	04:01,16:02	04:04,07:01	03:01:01G,04:01:01G	02:02,04:02
**10**	W054K163	01:01,02:01	15:08,40:06	01:02,15:02	06:03,06:03	13:01,13:01	04:01,09:01

Table S6: Information of donors in CRS assay

**Effector cells**		**Monocytes**
	Donor 7	Donor 7
**Effector T cells**	Donor 8	Donor 8
	Donor 9	Donor 9
**mExNK cells**	Donor 1	Donor 7
	Donor 2	Donor 8
	Donor 3	Donor 9
**CIML NK & PBNK cells**	Donor 4	Donor 7
	Donor 5	Donor 8
	Donor 6	Donor 9

Table S7: Information of donors in GvHD assay

**Effector cells**		**Allogenic PHA-blasts**	**Autologous PHA-blasts**
	Donor 7	Donor 10	Donor 7
**Effector T cells**	Donor 8	Donor 10	Donor 8
	Donor 9	Donor 10	Donor 9
**mExNK cells**	Donor 1	Donor 10	Donor 1

	Donor 2	Donor 10	Donor 2
	Donor 3	Donor 10	Donor 3
**CIML NK & PBNK cells**	Donor 4	Donor 10	Donor 4
	Donor 5	Donor 10	Donor 5
	Donor 6	Donor 10	Donor 6
